# Acceleration of Convergence to Equilibrium in Markov Chains by Breaking Detailed Balance

**DOI:** 10.1007/s10955-017-1805-z

**Published:** 2017-05-18

**Authors:** Marcus Kaiser, Robert L. Jack, Johannes Zimmer

**Affiliations:** 1 Department of Mathematical Sciences, University of Bath, Bath, BA2 7AY UK; 2 Department of Physics, University of Bath, Bath, BA2 7AY UK

**Keywords:** Convergence to equilibrium, Non-equilibrium processes, Zero-range process, Macroscopic Fluctuation Theory, Large deviations

## Abstract

We analyse and interpret the effects of breaking detailed balance on the convergence to equilibrium of conservative interacting particle systems and their hydrodynamic scaling limits. For finite systems of interacting particles, we review existing results showing that irreversible processes converge faster to their steady state than reversible ones. We show how this behaviour appears in the hydrodynamic limit of such processes, as described by macroscopic fluctuation theory, and we provide a quantitative expression for the acceleration of convergence in this setting. We give a geometrical interpretation of this acceleration, in terms of currents that are *antisymmetric* under time-reversal and orthogonal to the free energy gradient, which act to drive the system away from states where (reversible) gradient-descent dynamics result in slow convergence to equilibrium.

## Introduction

In this paper we analyse the effects of breaking detailed balance for interacting particle systems (as described by Markov processes [32]), and their hydrodynamic scaling limits (as described by Macroscopic Fluctuation Theory [9]). The interacting particle systems represent microscopic descriptions of physical systems, in which the motion of each particle may be followed individually. The (fluctuating) hydrodynamic model of the same system describes its behaviour on large length and time scales, in which case the motion of the individual particles is no longer visible, and one works instead with a smooth density field, whose time evolution includes a deterministic element as well as a (weak) stochastic noise [28].

Among interacting particle systems, those with detailed balance are special—they correspond to Markov chains that are reversible with respect to an invariant measure $\pi$. Physically, these models are important because their steady states are time-reversal symmetric and lack any persistent currents, so they can be used to describe systems that relax to states of thermal equilibrium. They also have applications outside physics, because given a (possibly non-normalised) measure $\nu$, it is straightforward to design a reversible Markov chain whose invariant measure $\pi$ is proportional to $\nu$. This construction is at the root of many Markov chain Monte Carlo (MCMC) methods [2, 34], in which one typically aims to generate large numbers of uncorrelated samples from a prescribed distribution $\pi$. Such methods have widespread applications including Bayesian learning, protein folding and cryptography [14].

In both the physical systems and the MCMC methods, an important question is the rate of convergence to equilibrium of the relevant Markov chains. In MCMC, this rate controls the computational cost required to obtain independent samples from $\pi$, which is an important factor in the efficiency of the method. In the physical systems, the question of how fast a system converges to equilibrium controls many physical properties including fluid viscosities, and systems’ abilities to respond to changes in external conditions, such as temperature.

Recently, several results have become available which show that for a given invariant measure $\pi$, reversible Markov chains have the slowest convergence [10, 24, 30, 35, 36, 39]. Given that most common MCMC methods are based on such reversible models, and that faster convergence is linked to improved efficiency, this observation offers a route towards the development of new and more efficient methods, some of which are already becoming available [5]. Breaking reversibility can be achieved by an explicit modification of transition rates [36], or by an expansion of the state space (*lifting*) to incorporate persistence of motion or inertial effects [12, 15]. The main physical feature of the resulting irreversible Markov chains is that they (generically) have non-equilibrium steady states characterised by finite entropy production and dissipation of energy. Compared to the equilibrium setting, the nature of fluctuations and convergence to steady state in non-equilibrium systems is much less understood, and is an area of important current activity [3, 9, 13].

To address these questions, this paper presents several new results. First, we revisit existing results for microscopic models, concentrating in particular on the spectral gap of the generator, and how it is affected when detailed balance is broken. Second, we investigate how breaking detailed balance affects the hydrodynamic limit of the model—in this latter case, convergence to equilibrium is most easily analysed via large deviations of the empirical measure [35, 36]. Third, we illustrate our general results by numerical results of a simple interacting particle system—the zero-range process [38]. These numerical results are particularly relevant since the analytical results indicate that breaking detailed balance can never slow down convergence to equilibrium, but they provide rather little insight into how much this convergence can be accelerated, nor how this effect depends on the specific way in which detailed balance is broken. We provide some general remarks and comments in this direction.

### Characterisation of Convergence to Steady State

A number of methods are available to analyse the time required for a system to reach its steady state. This section contains a brief review of some of them. For microscopic models—such as Markov processes on (finite) discrete spaces and SDEs—we mention some recent work showing how breaking detailed balance can accelerate convergence of systems to their steady states. These results serve as a foundation for our results here, which show how these effects manifest on the macroscopic scale.

#### Spectral Gap

The first—and most common—method for analysis of convergence to equilibrium is to estimate the spectral gap of the generator of the relevant stochastic process. In general, the eigenvalues $\{\lambda _i\}$ of the generator are complex numbers, there is a simple eigenvalue $\lambda _0=0$ and all other eigenvalues have negative real parts. The spectral gap $\alpha _\mathrm{min}$ is the minimal value of $|\lambda _i^r|$ among the non-zero eigenvalues, where $\lambda _i^r$ denotes the real part of $\lambda _i$. Roughly speaking, the physical significance of the spectral gap is that the system converges exponentially fast to its steady state, with a characteristic time scale

1 $$\begin{aligned} \tau _\mathrm{g}=(1/\alpha _\mathrm{min}). \end{aligned}$$

For stochastic differential equations [24, 30] and discrete-space Markov processes [36], it has been shown that irreversible processes generically have smaller time scales $\tau _\mathrm{g}$, compared to reversible processes with the same invariant measure. We provide further results in this direction in Sect. 2.1.1 below, for the discrete space Markov processes that are relevant for interacting particle systems.

#### Asymptotic Variance

Another set of methods for the analysis of the convergence to steady state is based on empirical time averages. That is, let $X_t$ be the state of the system at time *t* and let *f* be an observable quantity (test function) whose value at time *t* is $f(X_t)$. Then the empirical time average of *f* is

2 $$\begin{aligned} \overline{f}(T) := \frac{1}{T} \int _0^T f(X_s) \;\! ds. \end{aligned}$$

The quantity $\overline{f}(T)$ is a random variable which—under suitable conditions related to ergodicity—converges almost surely to the expectation value of *f*, which we denote by $\mathbb {E}_\pi (f)$.

Moreover the distribution of $\sqrt{T} (\bar{f}(T)-\mathbb E_\pi (f))$ converges by the central limit theorem to a normal distribution with variance $\sigma ^2_f$. The latter is referred to as asymptotic variance or time average variance constant (TAVC) which can be obtained as $\sigma ^2_f=\lim _{T\rightarrow \infty } T\mathrm {Var}(\bar{f}(T))$, see in [2, Chapter IV], [35] and [40, Sect. 3.5]. Hence, the variance of $\overline{f}(T)$ decays for large times as $\mathrm {Var}(\overline{f}(T))\sim \sigma ^2_f/T$. It is then natural to identify a time scale $\tau ^f_\mathrm{v}:=\sigma ^2_f/\mathrm {Var}_\pi (f)$. Note that $\tau ^f_\mathrm{v}$ depends on the observable *f* of interest—roughly speaking it represents the autocorrelation time of $f(X_t)$. In general $\tau ^f_\mathrm{v}$ and $\tau _\mathrm{g}$ are different time scales: $\tau ^f_\mathrm{v}$ controls the convergence of $\overline{f}(T)$ while $\tau _\mathrm{g}$ controls the convergence of the probability measure itself. As for $\tau _\mathrm{g}$, one finds that $\sigma ^2_f$ can be reduced by breaking detailed balance in Markov chains [10, 39] and SDEs [18, 25].

#### Large Deviations at Level-1 and Level-2

A more detailed analysis of the large-*T* behaviour of $\overline{f}(T)$ is available from large deviation theory [23, 40]. Informally, one expects that for large *T*, the random variable $\overline{f}(T)$ satisfies

3 $$\begin{aligned} \mathrm {Prob}\Bigl [ \overline{f}(T) \approx \hat{f}\Bigr ] \asymp \mathrm{e}^{-T I_f(\hat{f})} \end{aligned}$$

for some *rate function* $I_f$ (which depends on the choice of test function *f*). We use the notation in (3) throughout this work as an informal way to state large deviation principles: it means that the log probability that $\overline{f}(T)$ takes a value in a small interval containing $\hat{f}$ can be bounded above and below by quantities related to the rate function $I_f$ [23, 40]. The rate function achieves its minimal value of zero when $\hat{f}$ is equal to $\mathbb {E}_\pi (f)$, and the second derivative of $I_f$ at this minimum is related to $\sigma ^2_f$. The function $I_f$ is a level-1 rate function [23].

A yet more detailed analysis is available by considering not just the large deviations of a single test function *f* but instead to consider large deviations of the empirical measure. That is, for a Markov chain on a discrete space $\Omega$, define the empirical measure

4 $$\begin{aligned} \bar{\mu }_T(x) := \frac{1}{T} \int _0^T \delta _{X_s,x} \;\! ds , \end{aligned}$$

where $\delta _{x,y}$ is a Kronecker delta. The empirical measure at time *t* is a vector $\bar{\mu }_T = (\bar{\mu }_T(x))_{x\in \Omega }$. For large enough *T*, ergodicity implies that $\bar{\mu }_T(x)$ converges almost surely to $\pi (x)$, and the fluctuations of the measure $\mu$ in this limit are described by a large deviation principle at level-2:

5 $$\begin{aligned} \mathrm {Prob}\bigl [\bar{\mu }_T \approx \nu \bigr ] \asymp \mathrm{e}^{-T I_2(\nu )} \end{aligned}$$

where $I_2$ is the rate function [17], which now depends on a vector $\nu$ instead of a single real argument $\hat{f}$. Note that the (level-1) rate function $I_f$ for any observable *f* can be obtained by a contraction of this large deviation principle, so the function $I_2$ contains a great deal of information about the convergence of a system to its steady state. Moreover, as might be expected from the terminology “rate function”, the quantity $1/I_2(\mu )$ has an interpretation as a $\mu$-dependent time scale associated with the decay of an initial measure $\mu$ to the invariant measure $\pi$.

Recent work by Rey-Bellet and Spiliopoulos [35, 36] has motivated the analysis of $I_2$ as a measure of the rate of convergence of processes to their steady states. Their work, and that of Bierkens [10], show that breaking detailed balance accelerates this convergence. Note however that in contrast to the spectral gap—where a single number characterises the rate of convergence of the whole system—the rate function $I_2$ depends on the measure $\mu$ for which it is evaluated; similarly the asymptotic variance $\sigma ^2_f$ depends on the specific observable *f*. In this sense, the information available from the asymptotic variance and the large deviations is greater than that available from the spectral gap, but this extra information may also make these measures harder to interpret in terms of simple acceleration or slowing down of convergence to equilibrium. Of course, other useful measurements of convergence rates are available, such as mixing times [31], cutoff phenomena (see e.g. [29], where cutoff was recently established for the asymmetric simple exclusion process) and log-Sobolev constants (e.g. [16]), but these are not analysed in this work.

### Outline

The remainder of this paper is organised as follows: Sect. 2 includes a theoretical analysis of the effects of breaking detailed balance on convergence to steady states, including both Markov chains (Sect. 2.1) and hydrodynamic limits (Sect. 2.2). Section 3 presents numerical results that illustrate this acceleration in the zero-range process: we provides examples in both one-dimensional and two-dimensional settings. Finally, Sect. 4 contains our conclusions.

## Theoretical Results

### Acceleration of the Microscopic Dynamics

In this section, we consider an irreducible Markov jump process on a finite state space $\Omega$ which contains *n* states. In terms of interacting particle systems, this process describes the dynamics of a finite number of particles that move on some finite lattice. The process is defined by rates $c(x\rightarrow y)$ for states $x,y\in \Omega$. The condition of *detailed balance* (or *reversibility*) is that for some probability measure $\pi$ and all *x*, *y* then

6 $$\begin{aligned} \pi (x) c(x\rightarrow y) = \pi (y)c(y\rightarrow x) . \end{aligned}$$

In this case the (unique) invariant measure of the Markov process is $\pi$.

Let the generator of the Markov process be $\mathcal {L}$. The generator has a representation as an $n\times n$ matrix and the reversibility condition (6) corresponds to symmetry of $\mathcal {L}$ with respect to the $L^2(\pi )$ inner product $\langle f,g\rangle _{\pi }=\sum _x f(x) g(x) \pi (x)$. If detailed balance is broken (non-reversible Markov chain), then $\mathcal {L}$ is not symmetric with respect to $L^2(\pi )$, but one may always write

7 $$\begin{aligned} \mathcal {L}= \mathcal {L}_S + \mathcal {L}_A, \end{aligned}$$

where $\mathcal {L}_S$ is symmetric with respect to $L^2(\pi )$, while $\mathcal {L}_A$ is antisymmetric. Moreover, $\mathcal {L}_S$ is a generator for a reversible stochastic process, whose transition rates may be verified to be

8 $$\begin{aligned} c_s(x\rightarrow y) = \frac{1}{2} \left[ c(x\rightarrow y) + \pi (y) c(y\rightarrow x) \pi (x)^{-1} \right] , \end{aligned}$$

where $\pi$ is the invariant measure of $\mathcal {L}$, which is also the invariant measure of $\mathcal {L}_S$. (Recall that the original Markov process is finite and irreducible, which ensures that $\pi (x)>0$ for all *x*). We also identify the off-diagonal elements of $\mathcal{L}_A$ as

$$\begin{aligned} c_a(x\rightarrow y):=c(x\rightarrow y)-c_s(x\rightarrow y). \end{aligned}$$

Hence one has from (8) that

9 $$\begin{aligned} \pi (x)[c_s(x\rightarrow y) + c_a(x\rightarrow y)]=\pi (y)[c_s(y\rightarrow x) - c_a(y\rightarrow x)] . \end{aligned}$$

Note that $\mathcal L$ and $\mathcal L_S$ both are generators, whereas the operator $\mathcal L_A$ is not a generator of a Markov chain.

Alternatively one can think of the decomposition of $\mathcal L$ in $\mathcal L_S$ and $\mathcal L_A$ as follows: consider the Markov process $\eta _t$ (with $t\in [-T,T]$ for some $T>0$) associated to $\mathcal L$, with the initial condition distributed by the steady state $\pi$. The time reversed process $\hat{\eta }(t):=\eta (-t)$ is also associated to a generator, $\mathcal L^*$, say. The symmetric part of the generator can be recovered as $\mathcal L_S = (\mathcal L + \mathcal L^*)/2$.

Given these preliminaries, we can now be precise about the sense in which breaking detailed balance accelerates convergence: in all cases we compare the process $\mathcal {L}$ with the corresponding symmetrised process $\mathcal {L}_S$. (Equivalently, one may imagine starting from a reversible process $\mathcal {L}_S$ and breaking detailed balance by adding an extra term $\mathcal {L}_A$ to the generator.) The processes $\mathcal {L}$ and $\mathcal {L}_S$ both converge to the same invariant measure $\pi$—one aims to prove that convergence times such as $\tau _\mathrm{g}$ or $1/I(\mu )$ are smaller for $\mathcal {L}$ than for $\mathcal {L}_S$.

#### The Spectral Gap

To illustrate how breaking detailed balance accelerates convergence, we show in Proposition 1 below that breaking detailed balance can only increase the spectral gap, so that the convergence of the irreversible process is characterised by a smaller value of the time $\tau _\mathrm{g}$. This result has been proven in greater generality in [24, 36], but we provide a short proof here, for illustrative purposes.

To this end, consider an initial measure $\mu _0$, and represent it in terms of an eigendecomposition of $\mathcal {L}$, so that

10 $$\begin{aligned} \mu _0(x) = \pi (x) + \sum _{j=1}^m \bigl (\alpha _j\nu _j(x) + \bar{\alpha }_j \bar{\nu }_j(x)\bigr ), \end{aligned}$$

where the $\alpha _j\in \mathbb C$ are $\mu _0$-dependent coefficients, while $\nu _j$ are complex-valued measures which are left-eigenvectors corresponding to eigenvalues $\lambda _j$ of $\mathcal {L}$. The overbar (e.g. $\bar{\alpha }$) denotes the complex conjugate. Decomposing the non-zero eigenvalues $\lambda _j$ into real and imaginary parts, as $\lambda _j=\lambda _j^r+i\lambda _j^i$, the measure at time *t* is given by

11 $$\begin{aligned} \mu _t = (\mathrm e^{t\mathcal {L}})^\dagger \mu _0 = \pi + \sum _{j=1}^m \mathrm e^{\lambda _j^rt}\bigl (\mathrm e^{i\lambda _j^i t}\alpha _j\nu _j + \mathrm e^{-i\lambda _j^i t}\bar{\alpha }_j\bar{\nu }_j\bigr ), \end{aligned}$$

where $\cdot ^\dagger$ denotes a matrix transpose. Note that for real-valued eigenvalues (with $\lambda _j^i=0$) the term in brackets is equal to $2\alpha _j \nu _j$, as in this case also the left (and right) eigenvectors are real-valued.

Moreover, $\lambda _j^r<0$ for all *j*, since $\mathcal {L}$ is the generator of an irreducible finite Markov process. One sees immediately that this Markov process relaxes exponentially fast to its steady state. Moreover, the rate of this exponential decay is controlled by the non-zero eigenvalue of $\mathcal {L}$ whose real part is smallest in magnitude. Similar results to the following proposition have already been obtained in e.g. [26, 37]:

##### Proposition 1

Let $\mathcal L$ and $\mathcal L_S$ be given as above. The non-zero eigenvalues of $-\mathcal {L}_S$ are real and positive; let the smallest such eigenvalue be $\alpha _\mathrm{min}$ and the largest be $\alpha _\mathrm{max}$. Then every non-zero eigenvalue $\lambda$ of $-\mathcal {L}$ satisfies

12 $$\begin{aligned} \alpha _\mathrm{min} \le \mathrm{Re}(\lambda ) \le \alpha _\mathrm{max}. \end{aligned}$$

##### Proof

Define the Dirichlet form for $\mathcal {L}$ as $\mathcal E(f,g):= \langle f,-\mathcal L g\rangle _\pi = \sum _x f(x) \mathcal (-\mathcal Lg)(x)\;\!\pi (x)$, where $\pi$ is the unique stationary distribution of $\mathcal L$. Let $\lambda$ be a non-zero eigenvalue of $-\mathcal L$ with corresponding right eigenvector $f_\lambda +ig_\lambda$. As $\mathcal E(1,f)=0$ for all *f*, we obtain $0=\mathcal E(1,f_\lambda + i g_\lambda ) = \lambda (\langle 1,f_\lambda \rangle _\pi + i \langle 1,g_\lambda \rangle _\pi )$. Since $\lambda$ is non-zero, we obtain that $\langle 1,f_\lambda \rangle _\pi = 0 = \langle 1,g_\lambda \rangle _\pi$. This implies that both $f_\lambda$ and $g_\lambda$ are mean zero, so $\mathrm {Var}_\pi (h) = \langle h,h\rangle _\pi$ for $h\in \{f_\lambda ,g_\lambda \}$. Since $\mathcal E(f_\lambda -ig_\lambda , f_\lambda +ig_\lambda )= \lambda \langle f_\lambda -ig_\lambda ,f_\lambda +ig_\lambda \rangle _\pi = \lambda (\langle f_\lambda ,f_\lambda \rangle _\pi + \langle g_\lambda ,g_\lambda \rangle _\pi )$, the bilinearity of the Dirichlet form yields that the real part of $\lambda$ is given by

13 $$\begin{aligned} {\text {Re}}(\lambda ) = \frac{\mathcal E(f_\lambda ,f_\lambda ) + \mathcal E(g_\lambda ,g_\lambda )}{\langle f_\lambda ,f_\lambda \rangle _\pi + \langle g_\lambda ,g_\lambda \rangle _\pi }. \end{aligned}$$

In addition, one has (for the min and max taken over the two cases $h=f_\lambda$ and $h=g_\lambda$)

14 $$\begin{aligned} \min _{h\in \{f_\lambda ,g_\lambda \}}\frac{\mathcal E(h,h)}{\langle h,h\rangle _\pi } \le \frac{\mathcal E(f_\lambda ,f_\lambda ) + \mathcal E(g_\lambda ,g_\lambda )}{\langle f_\lambda ,f_\lambda \rangle _\pi + \langle g_\lambda ,g_\lambda \rangle _\pi } \le \max _{h\in \{f_\lambda ,g_\lambda \}}\frac{\mathcal E(h,h)}{\langle h,h\rangle _\pi }. \end{aligned}$$

Define $\mathcal{E}_S(f,g)=\langle f,-\mathcal L_S g\rangle _\pi$, and note that $\mathcal{E}(h,h)=\mathcal{E}_S(h,h)$. Also $\alpha _\mathrm{min}=\mathrm {min}_{h: \langle 1,h\rangle _\pi = 0} \frac{\mathcal E_S(h,h)}{\langle h,h\rangle _\pi }$ . Hence the left hand side of (14) is bounded below by $\alpha _\mathrm{min}$. Applying a similar argument to the right hand side of (14) and combining with (13) finally yields (12).

$$\square $$

#### Bounds on Level-2 Rate Functions for Discrete Markov Processes

From Proposition 1 and using (1), one clearly has

15 $$\begin{aligned} \tau _\mathrm{g}^\mathrm{irr} \le \tau ^\mathrm{rev}_\mathrm{g}. \end{aligned}$$

That is, the irreversible process converges to its steady state at least as quickly as the reversible one. A similar argument [10] establishes that the level-2 rate functions for $\mathcal {L}$ and $\mathcal {L}_S$ are related as

16 $$\begin{aligned} I_2(\mu ) \ge I_2^S(\mu ) , \end{aligned}$$

again establishing a faster rate of convergence on breaking detailed balance. Recall that results of the form (16) yield information about the empirical measure $\bar{\mu }_T$ defined in (4), whereas the previous result (12) concerns the spectral gap and the convergence of $\mu _t$, the distribution of the process at time *t* as defined in (11). Note that $\bar{\mu }_T$ is a random quantity, whereas $\mu _t$ is the solution to a deterministic differential equation.

We now show (Proposition 2) that the rate of convergence of the irreversible model has an upper bound, as well as the lower bound given by $I_2^S(\mu )$. That is, $I_2(\mu )$ is bounded both above and below, just as the spectral gap is bounded in (12). This limits the acceleration that is available by breaking detailed balance for (finite) discrete Markov processes, in contrast to the situation for diffusions [35]. The proof for the following proposition is based on the variational formula for the level-2 LDP [23]. Whilst the lower bound, which is known in the literature, see e.g. [10, 36], follows from the variational representation of the rate function, the upper bound is (to our knowledge) a novel result.

##### Proposition 2

Consider a finite-state continuous-time Markov chain with generator $\mathcal L = \mathcal L_S +\mathcal L_A$ and transition rates $c(x\rightarrow y)=c_s(x\rightarrow y)+c_a(x\rightarrow y)$, as defined in Sect. 2.1. The level-2 rate functional $I_2(\mu )$ is bounded as follows:

17 $$\begin{aligned} I_2^S(\mu ) \,{\le }\, I_2(\mu ) \,{\le }\, I_2^S(\mu ) \,{+}\, \sum _{x \not = y}\bigl [c_s(x\rightarrow y) - \sqrt{c_s(x\rightarrow y)^2-c_a(x\rightarrow y)^2}\bigr ] \,\sqrt{\tfrac{\mu (x)}{\pi (x)}\tfrac{\mu (y)}{\pi (y)}}\pi (x),\qquad \end{aligned}$$

where the rate functional $I_2^S(\mu )$ for the reversible process with generator $\mathcal L_S$ is given by $I_2^S(\mu ) = \bigl \langle \sqrt{\tfrac{\mu }{\pi }},- \mathcal L_S \sqrt{\tfrac{\mu }{\pi }}\bigr \rangle _\pi$.

##### Proof

The rate functional is given by a variational formula [23]:

$$\begin{aligned} I_2(\mu ) = \sup _{f>0}\;\! \langle f^{-1}, -\mathcal Lf\rangle _\mu . \end{aligned}$$

In the symmetric case ($\mathcal L=\mathcal L_S$) the maximum is $I_2^S(\mu )$, which is attained when $f=\sqrt{\mu /\pi }$. In general we write $f=\sqrt{\mu /\pi }\;\!\mathrm e^{V}$ for some potential *V*.

A direct computation yields

18 $$\begin{aligned} I_2^S(\mu ) = \sum \limits _{x \ne y} {\left( {\sqrt{\frac{{\mu (x)}}{{\pi (x)}}} - \sqrt{\frac{{\mu (y)}}{{\pi (y)}}} } \right) } \sqrt{\mu (x)\pi (x)} c(x \rightarrow y) \end{aligned}$$

and

19 $$\begin{aligned} {I_2}(\mu ) = I_2^S(\mu ) + \mathop {\sup }\limits _V {I_A}(\mu ,V) \end{aligned}$$

with

20 $$\begin{aligned} {I_A}(\mu ,V) = \sum \limits _{x \ne y} {\sqrt{\frac{{\mu (y)}}{{\pi (y)}}} } \left( {1 - {\mathrm{{e}}^{V(y) - V(x)}}} \right) \sqrt{\mu (x)\pi (x)} c(x \rightarrow y). \end{aligned}$$

If *V* is a constant function, then $I_A(\mu ,V)=0$ so clearly $\sup _V I_A(\mu ,V)\ge 0$. Hence, (19) yields the lower bound in (17), as in [10].

For the upper bound, it is convenient to define $m(x,y):=\frac{1}{2}\sqrt{\frac{\mu (x)\mu (y)}{\pi (x)\pi (y)}}$ and $q(x,y):=\pi (x)c(x\rightarrow y)$. This yields

21 $$\begin{aligned} I_A(\mu ,V) = \sum _{x\not =y} m(x,y) \left[ (1-\mathrm e^{V(y)-V(x)})q(x,y) + (1-\mathrm e^{V(x)-V(y)})q(y,x)\right] , \end{aligned}$$

where we have symmetrised the summand with respect to *x*, *y*. For positive constants *a*, *b*, one may easily establish the general inequality $a\mathrm{e}^{V} + b\mathrm{e}^{-V} \ge 2\sqrt{ab}$. Applying this inequality to the summand in (21) yields

22 $$\begin{aligned} I_A(\mu ,V) \le \sum _{x\not =y} m(x,y) \left[ q(x,y)+q(y,x) - 2\sqrt{q(x,y)q(y,x)} \right] . \end{aligned}$$

From (8), (9) one has $q(x,y)+q(y,x)=2c_s(x\rightarrow y)\pi (x)$ and $q(x,y)q(y,x)=[c_s^2(x\rightarrow y)-c_a^2(x\rightarrow y)]\pi (x)^2$; substituting these results into (22) yields

$$\begin{aligned} I_A(\mu ,V) \le \sum _{x\not =y}\sqrt{\tfrac{\mu (x)}{\pi (x)}\tfrac{\mu (y)}{\pi (y)}} \bigl [c_s(x\rightarrow y) - \sqrt{c_s(x\rightarrow y)^2-c_a(x\rightarrow y)^2}\bigr ]\pi (x), \end{aligned}$$

and the combination with (19) establishes the upper bound in (17).$\square$

#### Discussion

Our intuition for the (bounded) acceleration by breaking detailed balance is as follows: for reversible processes we can think of $\mu _t$ (the distribution of the process at time *t*) undergoing a steepest descent process (gradient flow) for the free energy $F(t)=\sum _x \mu _t(x) \log ( \mu _t(x)/\pi (x))$, within a particular geometric setting [33]. The precise nature of this geometry is immaterial for this discussion: the key point is that relaxation to equilibrium is fast when the free energy gradient is steep, and tends to be slow when it is shallow. On breaking detailed balance, the free energy still decreases monotonically, but its motion is no longer restricted to the direction of steepest descent. This can have several possible effects and the rate of change of *F*(*t*) may either increase or decrease on breaking detailed balance. However, we argue that an important contribution to the acceleration of convergence arises because the irreversible component of the dynamics drives the system away from regions where the free energy gradient is shallow and into regions where it is steeper. We will demonstrate this effect explicitly at the hydrodynamic level, in Sect. 2.2.3.

Notice however, that while slow processes associated with $\mathcal {L}_S$ are accelerated by breaking detailed balance, the inequality involving $\alpha _\mathrm{max}$ in Proposition 1 implies that fast aspects of the relaxation tend to be slowed down. Indeed, $\mathrm {tr}(\mathcal {L}_A)=0$ so $\mathrm {tr}(\mathcal {L})=\mathrm {tr}(\mathcal {L}_S)$: since the trace is equal to the sum of the eigenvalues, one sees that if some (slow) processes are accelerated by breaking detailed balance another set of (faster) processes must be slowed down by a similar amount. Within the intuitive picture, our interpretation is that the irreversible component of the dynamics acts to push the system away from regions where the free energy gradient is very steep, so the differences between very fast and very slow processes tend to be smoothed out by the irreversibility.

### Accelerating Macroscopic Processes

In this section we consider hydrodynamic limits of interacting particle systems, as described by the macroscopic fluctuation theory (MFT) [9]. We will demonstrate that the large deviation result (16) has a counterpart at the hydrodynamic level. We also explore the geometrical interpretation of this result, and we connect our result to earlier work related to SDEs that describe the motion of single particles [35].

#### Macroscopic Fluctuation Theory

We first recall the core parts of the macroscopic fluctuation theory (MFT). For a detailed review we refer to [9]. Let $\Lambda \subseteq \mathbb R^d$ be a connected domain with boundary $\partial \Lambda$. For simplicity, we choose here the domain $\Lambda =[0,1]^d$. If we consider a microscopic particle process (indexed by *L*), its description within MFT involves two random fields, the empirical particle density$\rho _t^L$ and the empirical current $j_t^L$. Roughly speaking, for $x\in \Lambda$ then $\rho _t^L$ is the local particle density and $j_t^L$ is a vector that indicates the rate of particle flow.

The idea of the hydrodynamic limit is that if we observe an interacting particle system on suitably large scales of length and time, then the system can be described in terms of sufficiently smooth fields $\rho$ and *j*, instead of requiring a microscopic description in which all particle positions are taken into account. The deterministic quantities $\rho$ and *j* are then related by a continuity equation given by

23 $$\begin{aligned} \partial _t \rho _t + \nabla \cdot j_t = 0 . \end{aligned}$$

The domain $\Lambda$ is fixed in the hydrodynamic limit. The relevance to large length and time scales in the microscopic model is that one considers a large number of particles *N* within a domain $\Lambda _L$ of linear size *L*. One takes *N*, *L* to infinity together for a fixed density $\tilde{\rho }_0=N/L^d$. The domain $\Lambda$ is obtained by rescaling the (increasingly large) domain $\Lambda _L$, so that $\Lambda$ remains fixed as $L\rightarrow \infty$.

Within this hydrodynamic limit, the behaviour of the system on suitably large scales of space and time becomes increasingly deterministic. For example, given a time interval [0, *T*] and initial and final densities $\rho _0$ and $\rho _T$, the probability measure for paths connecting these initial and final states concentrates (in the hydrodynamic limit) on a single most likely path. This result can be expressed as a large deviation principle for paths, which can, following [9], be written as

24 $$\begin{aligned} \mathrm {Prob}\left[ (\rho _t^L,j_t^L)_{t\in [0,T]} \approx (\rho _t,j_t)_{t\in [0,T]} \right] \asymp \mathrm{e}^{-L^d\mathcal{I} (\rho ,j)} \end{aligned}$$

with

25 $$\begin{aligned} \mathcal I(\rho , j) =\frac{1}{4}\int _{0}^{T}\int _\Lambda (j_t-J(\rho _t))\cdot \chi (\rho _t)^{-1}(j_t-J(\rho _t))\;\!dx\;\!dt \end{aligned}$$

whenever $\partial _t \rho _t = -\nabla \cdot j_t$ is satisfied, and $\mathcal I(\rho , j) =\infty$ otherwise. We refer the reader to the review [9] for details on the validity of (24) for a large class of particle systems including the symmetric exclusion process and zero-range processes [28, 38].

Note that in contrast to the large deviation principle in Sect. 1.1.3 which is concerned with large times, this principle involves a limit of large *L*, with a fixed time interval [0, *T*].

Physically, we interpret $J(\rho _t)$ in (25) as the most likely current field $j_t$, given that the system has density $\rho _t$. Within MFT, the current is assumed [9, Eq. (2.6)] to have the form

26 $$\begin{aligned} J(\rho ) = -D(\rho ) \nabla \rho + \chi (\rho ) E , \end{aligned}$$

where $\chi (\rho )$ and $D(\rho )$ are symmetric positive definite $d\times d$ matrices that depend on the local density $\rho$, and *E* is a fixed (*x*-dependent) vector field.

Physically, *D* and $\chi$ correspond to a density-dependent diffusivity and mobility, while *E* corresponds to an external force. For a given interacting particle system, the parameters *D*, $\chi$ and *E* can (in principle) be derived from the microscopic rules of the model. These parameters (along with appropriate boundary conditions associated with $\partial \Lambda$) fully specify the rate function (25) and they fully describe the hydrodynamic limit of the interacting particle system. To fix the ideas precisely, it may be useful to note that $J(\rho )$ in (26) is itself a field, whose value at position $x\in \Lambda$ is $J(\rho )(x) = -D(\rho (x)) \nabla \rho (x) + \chi (\rho (x)) E(x)$.

Since $J(\rho )$ is the most likely current for a given density $\rho$, it follows that for a given initial condition, the path measure is dominated by paths $(\rho _t)_{t\in [0,T]}$ which solve $\partial _t \rho = - \nabla \cdot J(\rho )$. These paths have $\mathcal{I}=0$ and are said to satisfy the hydrodynamics.

As well as the large-deviation principle for paths (24), the MFT also provides a large-deviation principle for the fluctuations of the instantaneous density, in the steady state of the system. That is, if the time *T* is large enough that the system has converged to its steady state, one has

27 $$\begin{aligned} \mathrm {Prob}[\rho _T^L \approx \rho ] \asymp \mathrm{e}^{-L^d\mathcal{V}(\rho )}, \end{aligned}$$

where $\mathcal V$ is called the quasipotential: it determines the probability of fluctuations in the density. Eq. (27) is derived under the assumption that the adjoint dynamics satisfy a further Large Deviation principle for a rate functional $\mathcal I^*$. We refer to chapter II in [9] for a detailed discussion.

We assume throughout that our system has a unique steady state, for which the most likely (*x*-dependent) density is $\overline{\rho }$. In this case $\mathcal{V}(\overline{\rho })=0$ and $\mathcal{V}(\rho )>0$ for all $\rho \ne \overline{\rho }$.

#### Reversible and Irreversible Systems

For the microscopic dynamics, we already observed that the detailed balance condition (6) describes an important special case. By starting from this case, the generator was decomposed into two components (7), corresponding to a reversible process and a correction term that captures the irreversibility. At the hydrodynamic level, there is a corresponding decomposition which takes place at the level of the current: one writes

28 $$\begin{aligned} J=J_S + J_A . \end{aligned}$$

The symmetric part of the current is defined [9, Equ. (2.19)] as

29 $$\begin{aligned} J_S(\rho ) = - \chi (\rho ) \nabla \frac{\delta \mathcal{V}}{\delta \rho }, \end{aligned}$$

where $\frac{\delta \mathcal{V}}{\delta \rho }$ denotes the functional derivative of the quasipotential introduced in Eq. (27). The antisymmetric part of the current is orthogonal to $J_S$, in the sense that

30 $$\begin{aligned} \int _\Lambda J_A(\rho ) \cdot \chi ^{-1}(\rho ) J_S(\rho ) \;\! dx = 0 , \end{aligned}$$

which is sometimes referred to as a *Hamilton-Jacobi equation*. Note that this is an orthogonality in the space of fields: the presence of the integral implies that the currents $J_S$ and $J_A$ do not have to be orthogonal at any specific point *x*. We note that on combining (29) and (30), one has $\int _\Lambda J_A(\rho ) \cdot \nabla \frac{\delta \mathcal{V}}{\delta \rho } \;\! dx = 0 ;$ integrating by parts and using (28) one sees that

31 $$\begin{aligned} \partial _t \mathcal{V} = \langle \partial _t \rho , \frac{\delta \mathcal{V}}{\delta \rho } \rangle = \langle \mathrm{div} J, -\frac{\delta \mathcal{V}}{\delta \rho } \rangle = -\langle J_S,\chi ^{-1} J_S\rangle \end{aligned}$$

which is independent of $J_A$. Hence the quasipotential is non-increasing for paths satisfying the hydrodynamics, and (for any given $\rho _t$) its time derivative is independent of $J_A$.

The special case in which the microscopic model is reversible has two implications for the hydrodynamic limit as described by MFT. First, reversible models lead to $J_A=0$, so $J=J_S$. Second, assuming that correlations in the particle model occur only on the microscopic scale, the quasipotential within the MFT takes the simple (local) form [9, Eq. (2.25)]

32 $$\begin{aligned} \mathcal{V}(\rho )=\int _\Lambda \Bigl [ f(\rho )-f(\overline{\rho })-f'(\overline{\rho })(\rho -\overline{\rho }) \Bigr ]\;\! dx, \end{aligned}$$

where $f(\rho )$ is the free energy per unit volume. (The dependence of *f* on $\rho$ is fixed by the microscopic model of interest; note also that both $\rho$ and $\overline{\rho }$ depend in general on the position *x*, but *f* is a local function $f(\rho )(x)=f(\rho (x))$).

Hence for reversible microscopic models, the hydrodynamic current obeys

33 $$\begin{aligned} J(\rho ) = J_S(\rho ) = -\chi (\rho ) f''(\rho ) \nabla \rho + \chi (\rho ) \nabla f'(\overline{\rho }) . \end{aligned}$$

In this case consistency with (26) requires

34 $$\begin{aligned} E=\nabla f'(\overline{\rho }), \qquad D(\rho )=f''(\rho )\chi (\rho ) . \end{aligned}$$

The second of these conditions is required within MFT. It is known as the local Einstein relation since it relates the mobility $\chi$ to the diffusion constant *D*. Note that the equations (34) are consistent with the hydrodynamic limit for a large class of particle systems of ‘gradient type’, see [9, Chap. VIII, Sect. G].

We end this section with a brief comment on the boundary conditions within MFT. If the boundary is associated with coupling of the system to a reservoir at chemical potential $\lambda$, the density at the boundary is fixed such that $f'(\rho )=\lambda$. If particles cannot penetrate the boundaries, one requires $D\nabla \rho =\chi E$ (and $j=0$) on $\partial \Lambda$. Paths (or configurations) that do not respect these boundary conditions have $\mathcal{I}=\infty$.

#### Breaking Detailed Balance Accelerates Convergence

We now state the sense in which breaking detailed balance accelerates convergence of interacting particle systems at the hydrodynamic scale. For the microscopic models, we compared two Markov chains, with the same invariant measure and generators $\mathcal {L}$ and $\mathcal {L}_S$. At the hydrodynamic scale, we will compare two systems with the same quasipotential (this corresponds to comparing two microscopic models with the same invariant measure). One system is irreversible and has a general *J* given by (26); the second system is reversible and so $J_A=0$. In order to ensure a fair comparison, we also assume that the two models have the same mobility $\chi (\rho )$: for Markov processes the equivalent condition was that we always compared models with the same $\mathcal{L}_S$. Since $\mathcal V$ and $\chi$ are the same for both models, they both have the same symmetric current $J_S$ which is given by (29).

For each of these systems, we consider the large deviations of the time-averaged density, following Sect. 1.1.3. Large deviation principles of the form

35 $$\begin{aligned} \mathrm {Prob}\left[ \frac{1}{T} \int _0^T \rho ^L_t(\cdot ) \;\!dt \approx \rho (\cdot ) \right] \asymp \mathrm{e}^{-T L^d I_2(\rho )} \end{aligned}$$

apply in both reversible and irreversible models. This large deviation principle applies on taking the large-*T* limit after the hydrodynamic limit: one should take $L\rightarrow \infty$ before $T\rightarrow \infty$. To obtain bounds on $I_2$, we introduce the so-called level-2.5 large-deviation principle for the joint fluctuations of the empirical current and empirical measure [4, 8]. That is,

36 $$\begin{aligned} \mathrm {Prob}\left[ \frac{1}{T} \int _0^T \rho ^L_t(\cdot )\;\!dt \approx \rho (\cdot ), \frac{1}{T} \int _0^T j^L_t(\cdot ) \;\!dt \approx j(\cdot )\right] \approx \mathrm{e}^{-T L^d I_{2.5}(\rho ,j)} . \end{aligned}$$

If we assume that the paths that dominate the level-2.5 LDP are constant in time, the relevant rate function can be obtained from (24) as

37 $$\begin{aligned} I_{2.5}(\rho ,j) = \frac{1}{4} \int _\Lambda (j-J(\rho ))\cdot \chi (\rho )^{-1}(j-J(\rho ))\;\!dx \end{aligned}$$

if $\nabla \cdot j=0$, and $I_{2.5}=\infty$ otherwise. The assumption of time-independent paths is equivalent to assuming that no dynamical phase transition takes place [6, 11]. Using this assumption, we now calculate a bound (Proposition 3) for the level-2 rate functionals, which is analogous to (16) in the microscopic case.

##### Proposition 3

Let the level-2.5 rate functional be given by (37) and let $I_2$ be the level-2 large deviation rate functional obtained from $I_{2.5}$ by contraction. We write $I_2^\mathrm{rev}$ for this rate functional if the current is symmetric, $J = J_S$, and we write $I_2^\mathrm{irrev}$ for the rate functional for the general case $J = J_S + J_A$ as in (48). Then

38 $$\begin{aligned} I_2^\mathrm{irrev}(\rho ) \ge I_2^\mathrm{rev}(\rho ). \end{aligned}$$

*Remark* Note that this result will be strengthened later. We will obtain in equation (51) an exact identity for $I_2^\mathrm{irrev}$ as the sum of $I_2^\mathrm{rev}$ and a non-negative quantity.

##### Proof

We write $I_2$ for $I_2^\mathrm{irrev}$. The rate functional at level-2 can be obtained by a contraction of the level-2.5 rate functional,

39 $$\begin{aligned} I_2(\rho )=\inf _{j:\nabla \cdot j=0}I_{2.5}(\rho ,j) . \end{aligned}$$

Note that $I_{2.5}(\rho ,j)$ as given in Eq. (37) is [using (30)] equal to the sum of the following three summands:

40 $$\begin{aligned}&\frac{1}{4} \int _\Lambda (j-J_S(\rho ))\cdot \chi (\rho )^{-1}(j-J_S(\rho ))\;\!dx\nonumber \\&\quad +\frac{1}{4} \int _\Lambda (j-J_A(\rho ))\cdot \chi (\rho )^{-1}(j-J_A(\rho ))\;\!dx -\frac{1}{4} \int _\Lambda j\cdot \chi (\rho )^{-1}j\;\!dx. \end{aligned}$$

The summand in the first line coincides with the symmetric rate functional $I_{2.5}^\mathrm{rev}(\rho ,j)$ and the second line is the part that corresponds to the anti-symmetric dynamics. Dropping the first summand in the second line (which is non-negative), we obtain

41 $$\begin{aligned} I_{2.5}(\rho ,j)\ge \frac{1}{4} \int _\Lambda (j-J_S(\rho ))\cdot \chi (\rho )^{-1}(j-J_S(\rho ))\;\!dx -\frac{1}{4} \int _\Lambda j\cdot \chi (\rho )^{-1}j\;\!dx. \end{aligned}$$

An expansion of the square shows that the right hand side is equal to

$$\begin{aligned} \frac{1}{4} \int _\Lambda J_S(\rho )\cdot \chi (\rho )^{-1}J_S(\rho )\;\!dx -\frac{1}{2} \int _\Lambda J_S(\rho )\cdot \chi (\rho )^{-1} j\;\!dx, \end{aligned}$$

and the last summand vanishes under the assumption that $\nabla \cdot j=0$, as by Eq. (29)

42 $$\begin{aligned} \int _\Lambda J_S(\rho )\cdot \chi (\rho )^{-1} j\;\!dx =- \int _\Lambda \nabla \frac{\delta \mathcal{V}}{\delta \rho }\cdot j\;\!dx= \int _\Lambda \frac{\delta \mathcal{V}}{\delta \rho }\nabla \cdot j\;\!dx =0. \end{aligned}$$

We obtain with (39) that

$$\begin{aligned} I_2(\rho )=\inf _{j:\nabla \cdot j=0}I_{2.5}(\rho ,j)\ge \frac{1}{4} \int _\Lambda J_S(\rho )\cdot \chi (\rho )^{-1}J_S(\rho )\;\!dx. \end{aligned}$$

To establish (39) we now show that the right hand side of this expression coincides with $I_2^\mathrm{rev}(\rho )$. Note that again for *j* such that $\nabla \cdot j=0$, by the same argument as in (42), the reversible level-2.5 rate functional is equal to

43 $$\begin{aligned} I_{2.5}^\mathrm{rev}(\rho ,j)= \frac{1}{4} \int _\Lambda j\cdot \chi (\rho )^{-1} j \;\! dx + \frac{1}{4} \int _\Lambda J_S(\rho )\cdot \chi (\rho )^{-1}J_S(\rho )\;\! dx. \end{aligned}$$

As one would expect for the reversible case, the infimum in (39) is clearly attained for a vanishing current ($j=0$), so that

44 $$\begin{aligned} I_2^\mathrm{rev}(\rho )= \frac{1}{4} \int _\Lambda J_S(\rho )\cdot \chi (\rho )^{-1}J_S(\rho )\;\!dx, \end{aligned}$$

which completes the proof. $\square$

Of course, given the acceleration at the microscopic scale, the result (38) that this acceleration is preserved at the hydrodynamic limit may not be surprising. However, we show below that the geometric structure underlying the MFT allows some stronger results for this acceleration to be established.

#### Splitting the Current

To understand the geometrical origin of (38) in more detail, we now show that as well as the decomposition (28), the antisymmetric current $J_A$ has a further decomposition into two parts which are orthogonal to each other, and are both orthogonal to $J_S$. [Here, orthogonality should be understood in the sense of (30).]

We consider the problem

45 $$\begin{aligned} \nabla \cdot \bigl (\chi (\rho ) \nabla \psi \bigr ) = -\nabla \cdot J_A(\rho ), \end{aligned}$$

with the boundary condition $\psi = 0$ on $\partial \Lambda$. For any fixed $\rho$ (such that $\chi (\rho )$ and $J_A(\rho )$ are sufficiently regular) Eq. (45) has a unique strong solution $\psi$ (see for example Theorem 6.24 in [20]). This solution $\psi$ is therefore a functional of $\rho$ we will denote with $\psi (\rho )$. Equation (45) motivates us to decompose $J_A(\rho )$ as

46 $$\begin{aligned} J_A(\rho ) = -\chi (\rho ) \nabla \psi (\rho ) + J_F(\rho ) , \end{aligned}$$

where $J_F(\rho )$ is a new vector field, which is again a functional of $\rho$. From (45) we see that

47 $$\begin{aligned} \nabla \cdot J_F(\rho ) = 0 \end{aligned}$$

for all $$\rho $$.

We arrive at the following structure for the hydrodynamic current:

48 $$\begin{aligned} J(\rho ) = J_S(\rho ) -\chi (\rho ) \nabla \psi (\rho ) + J_F(\rho ). \end{aligned}$$

Of the three terms on the right hand side, the first is familiar as the symmetric current, while the third is divergence free and so does not transport any density. The remaining term (involving $\psi$) specifies how the density is transported by the antisymmetric current, and also determines the large deviations at level-2. The latter will be established below as a consequence of the following proposition.

##### Proposition 4

The three terms on the right hand side of Eq. (48) are all orthogonal in the sense of Eq. (30). Moreover, $J_S(\rho )$ and $-\chi (\rho )\nabla \psi (\rho )$ are orthogonal to all divergence free vector fields that vanish on the boundary.

##### Proof

Consider first the orthogonality between $J_F(\rho )$ and $\chi (\rho )\nabla \psi (\rho )$. One has $\psi (\rho )|_{\partial \Lambda }=0$ so integration by parts yields

$$\begin{aligned} \int _\Lambda \chi (\rho )\nabla \psi (\rho ) \cdot \chi ^{-1}(\rho ) J_F(\rho ) \;\! dx = -\int _\Lambda \psi (\rho ) \nabla \cdot J_F(\rho ) \;\! dx = 0, \end{aligned}$$

where the second equality follows from (47). Hence $J_F(\rho )$ and $\chi (\rho )\nabla \psi (\rho )$ are orthogonal in the sense of (30).

Following the same method but replacing $\psi$ by $\delta \mathcal{V}/\delta \rho$ shows that $J_F(\rho )$ is orthogonal to $J_S(\rho )=-\chi (\rho )\nabla (\delta \mathcal{V}/\delta \rho )$, where we used $(\delta \mathcal{V}/\delta \rho )|_{\partial \Lambda }=0$, as discussed in [9].

Finally, using the orthogonality relation (30) and $J_A(\rho )=-\chi (\rho ) \nabla \psi (\rho ) + J_F(\rho )$ yields

$$\begin{aligned} \int _\Lambda \chi (\rho )\nabla \psi (\rho ) \cdot \chi ^{-1}(\rho ) J_S \;\! dx = \int _\Lambda J_F(\rho ) \cdot \chi ^{-1}(\rho ) J_S(\rho ) \;\! dx. \end{aligned}$$

The right hand side vanishes by orthogonality of $J_S(\rho )$ and $J_F(\rho )$, so $\chi (\rho ) \nabla \psi (\rho )$ is orthogonal to $J_S(\rho )$, as required. $\square$

Combining Eq. (48) and Eq. (47), the dynamics of the density is given by

49 $$\begin{aligned} \partial _t \rho = \nabla \cdot \bigl (\chi (\rho ) \bigl [\nabla \tfrac{\delta \mathcal V}{\delta \rho } + \nabla \psi (\rho )\bigr ]\bigr ). \end{aligned}$$

The first term on the right hand side describes steepest descent (gradient flow) of the quasipotential, within a (modified) Wasserstein metric [1, 27]. The second term describes a current that is orthogonal to the gradient flow (within the same metric), and leads to an evolution of $\rho$ within the level sets of the quasipotential: this is the geometric result anticipated in Sect. 2.1.3, but in this hydrodynamic setting the geometrical objects are more explicit.

We now derive exact formulas for the level-2.5 and level-2 rate functionals based on the splitting in Proposition 4.

##### Proposition 5

Let the level-2.5 large deviation rate functional be given by (37). Further let $\rho$ be such that Eq. (45) has a unique classic solution (up to a constant) and *j* such that $\nabla \cdot j= 0$. Then,

50 $$\begin{aligned} I_{2.5}(\rho ,j) =&\frac{1}{4} \int _\Lambda (j-J_F(\rho ))\cdot \chi (\rho )^{-1}(j-J_F(\rho ))\;\!dx\nonumber \\&+ \frac{1}{4} \int _\Lambda \nabla \tfrac{\delta \mathcal V}{\delta \rho }\cdot \chi (\rho ) \nabla \tfrac{\delta \mathcal V}{\delta \rho }\;\!dx + \frac{1}{4}\int _\Lambda \nabla \psi (\rho )\cdot \chi (\rho ) \nabla \psi (\rho )\;\!dx. \end{aligned}$$

Moreover, the level-2 rate functional is given by

51 $$\begin{aligned} I_2(\rho ) = \frac{1}{4} \int _\Lambda \nabla \tfrac{\delta \mathcal V}{\delta \rho }\cdot \chi (\rho ) \nabla \tfrac{\delta \mathcal V}{\delta \rho }\;\!dx + \frac{1}{4}\int _\Lambda \nabla \psi (\rho )\cdot \chi (\rho ) \nabla \psi (\rho )\;\!dx. \end{aligned}$$

##### Proof

The proof of equation (50) follows from Proposition 4 and the representation of the rate functional (40). The second result (51) follows readily as $j=J_F(\rho )$ is the minimiser of (50).$\square$

Note that these results are consistent with (43) and (44), where the minimising current was given by $j=0$. In the general case, the minimising current is given by $j=J_F(\rho )$.

We moreover can recognise the first term on the right hand side of (51) as $I_2^\mathrm{rev}(\rho )$, so the second term on the right hand side is an exact formula for the difference in rate for reversible and irreversible processes. This shows that the convergence rate for the irreversible process is strictly faster, unless the force $(-\nabla \psi )$ vanishes. We recognise this as a condition that the antisymmetric part of the current contributes to the time derivative of the density (otherwise the convergence to equilibrium of the density can not be accelerated).

Note that the objects $\nabla \tfrac{\delta \mathcal V}{\delta \rho }$ and $\nabla \psi$ should be interpreted as forces acting in the space of densities. In order to sustain a large deviation of the density, the stochastic forces within the system must act to resist these (deterministic) forces. One sees from (51) that the probability of this rare event (or large deviation) is given by the norms of the two forces, within a metric that depends on the mobility $\chi$.

#### An Example

We have discussed the status of the MFT as a theory for the hydrodynamic limit of interacting particle systems. For a concrete example of this approach, we consider an interacting particle model known as the zero-range process (ZRP) [38]. A microscopic description of the ZRP is given in Sect. 3.1. For the purposes of this section, the important features of the ZRP are that its hydrodynamic limit is described by the MFT and that irreversible ZRPs have local quasipotentials of the form (32). This latter fact allows straightforward comparison between reversible and irreversible models with the same quasipotential.

The hydrodynamic limit of the ZRP is a non-linear drift-diffusion

52 $$\begin{aligned} \partial _t \rho = \Delta \phi (\rho ) - \nabla \cdot \bigl (\phi (\rho )E\bigr ), \end{aligned}$$

where $\phi$ is a function that depends on the local density [that is, $\phi (\rho )(x)=\phi (\rho (x))$], and *E* is a drift term. The specific function $\phi$ that appears in the MFT depends on how the particles interact within the ZRP. A formal derivation of this hydrodynamic limit can e.g. be found in [9]. If $\phi (\rho )=\rho$, then the model corresponds to drift-diffusion of non-interacting particles.

One sees immediately from (52) that the hydrodynamic current is given by (26) with $\chi (\rho )=\phi (\rho )I$ and $D(\rho )=\phi '(\rho )I$, where *I* is the identity matrix. Moreover, the quasipotential for the ZRP is given by (32) with $f'(\rho )=\log \phi (\rho )$, consistent with (34). The ZRP may be either reversible or irreversible: one sees that reversible ZRPs lead to $E=-\nabla V$ for some potential *V*. In this case (34) shows that $V(x)=\log (\phi (\overline{\rho }(x)))+\lambda$, where $\overline{\rho }$ is the steady state density profile and $\lambda$ is a constant (independent of *x*). Hence one identifies the irreversible current as $J_A(\rho )=J(\rho )-J_S(\rho )=\phi (\rho ) \left[ E + \nabla \log \phi (\overline{\rho })\right]$.

Examining the rate function (51) for the specific case of the ZRP, one can interpret the result as a generalisation of a result in [35]. One has $\delta \mathcal{V}/\delta \rho = \log \phi (\rho ) - \log \phi (\overline{\rho })$. Hence

53 $$\begin{aligned} I_2(\rho ) =\int \Bigl (\bigl |\nabla \log \bigl (\tfrac{\phi (\rho )}{\phi (\bar{\rho })}\bigr )\bigr |^2 +|\nabla \psi (\rho )|^2\Bigr )\phi (\rho )\;\! dx, \end{aligned}$$

where $\psi$ is the solution of $\nabla \cdot \bigl (\phi (\rho ) \nabla \psi \bigr ) = -\nabla \cdot [ \phi (\rho ) ( E+\nabla \log \phi (\overline{\rho }))]$. If we now consider the special case $\phi (\rho )=\rho$ then we recover the same rate function as in Theorem 2.2 of Ref. [35]: the non-gradient force *C* in that work is here replaced by $E+\nabla \log \overline{\rho }$ (note that this is independent of $\rho$). The condition that $\nabla \cdot (\overline{\rho }C)=0$—which ensures that the invariant measure is unchanged by breaking detailed balance—is satisfied within the MFT because $\nabla \cdot J_A(\overline{\rho })=0$ and setting $\phi (\rho )=\rho$ yields $J_A(\overline{\rho })=\overline{\rho }(E+\nabla \log \overline{\rho })$.

Note however the setting discussed in this work is different to that in [35]: here we consider the hydrodynamic limit of many particles on a lattice while that work considers a single particle in a compact manifold without boundary. For non-interacting particles, the result is the same: the reason that for the many-particle system, the rate function $I^N$ associated with all the particles undergoing the same rare fluctuation is equal to $NI^{1}$. So the only difference between the one-particle and many-particle systems arises in the prefactors (speeds) of the large deviation principles (35), (36).

## Application to the Zero-Range Process, and Numerical Results

### The Zero-Range Process

The ZRP [38] is a system in which interacting particles move on a finite lattice $\Lambda _L=\{0,\dots ,L-1\}^d \subseteq \mathbb Z^d$ where $L\in \mathbb N$ is the linear system size. The particles are assumed to be indistinguishable and each particle is located at one of the sites $x\in \Lambda _L$. The number of particles on site *x* is $\eta (x)$ and the configurations of the system are $\eta =(\eta (x))_{x\in \Lambda _L}$. We will assume that the total number of particles is conserved such that no particles are added or removed over time.

The interaction of the particles is encoded in a function *g*(*k*), with $g(0)=0$. The rate of particle transfer from site *x* to site *y* is $g(\eta (x))c(x\!\rightarrow \!y)$, where the function *c* determines the connectivity of the sites. The case $g(k)=k$ corresponds to non-interacting particles. The model is referred to as zero-range because particles interact only when they are on the same site. For example, if $g(k)=k^\alpha$ for $k > 0$, then $\alpha <1$ means particles on the same site attract each other (suppressing jumps away from that site) while $\alpha >1$ means that particles on the same site tend to repel each other.

#### Reversible and Irreversible ZRP

The behaviour of the ZRP depends strongly on the choice of the connectivity function *c* as well as the interaction function *g*. We assume that particles hop only to nearest neighbour sites, so $c(x\rightarrow y)>0$ only if *x* and *y* are nearest neighbours. At the boundaries of the lattice, the system has either reflecting boundaries (particles cannot leave the lattice) or periodic boundaries.

It is easily verified that the model obeys the detailed balance condition (6) if one takes (for nearest neighbour sites)

54 $$\begin{aligned} c(x \rightarrow y) = \mathrm{e}^{\frac{1}{2}[V(x)-V(y)]} \end{aligned}$$

for some potential function *V*. In this case the model is reversible.

To arrive at a class of irreversible models, we take

55 $$\begin{aligned} c(x \rightarrow y) = \mathrm{e}^{\frac{1}{2}[V(x)-V(y)]} + k_{x,y} \mathrm{e}^{V(x)} \end{aligned}$$

with $$k_{x,y} =-k_{y,x} $$. In this case positivity of transition rates requires $$|k_{x,y}|<\mathrm{e}^{-\frac{1}{2}[V(x)+V(y)]}$$ for all *x*, *y*. We show below that taking $$k\ne 0$$ corresponds to breaking of detailed balance, in the sense of (7).

#### Generator and Invariant Measure

We denote the configuration of the ZRP at time *t* with $\eta _t$. The generator acts on the test function *f* as

56 $$\begin{aligned} \mathcal L f(\eta ) = \sum _{x,y\in \Lambda _L} (f(\eta ^{x,y})-f(\eta )) g(\eta (x))c(x\!\rightarrow \!y). \end{aligned}$$

Here $\eta ^{x,y}$ denotes the configuration obtained from $\eta$ by removing one particle from position *x* and adding it at position *y*. If $\eta (x)=0$ we simply set $\eta ^{x,y}=\eta$ and hence leave the configuration unchanged.

Note that the ZRP as defined so far is reducible, since the number of particles is a conserved quantity under the dynamics. This setting is useful because it is easily verified (directly from the definition (56) and using that the invariant measure $\pi$ satisfies $\sum _\eta \pi (\eta ) \mathcal {L}f(\eta )=0$ for all *f*) that the reversible model with rates defined in (54) has a family of invariant measures, the so called grand-canonical measures, which are parameterised by the chemical potential $\lambda$ and given by

57 $$\begin{aligned} \pi _{\mathrm {grand}}^\varphi (\eta ) = \prod _{x\in \Lambda _L} \frac{\varphi (x)^{\eta (x)}}{z(\varphi (x)) g!(\eta (x))} \end{aligned}$$

with the fugacity $\varphi (x)=\mathrm e^{-V(x)-\lambda }$ for some $\lambda \in \mathbb R$; the notation *g*!(*k*) indicates the generalised factorial $g!(k):=\prod _{i=1}^k g(i)$ [with $g!(0)=1$] and $z(\varphi )=\sum _{k=0}^\infty \frac{\varphi ^{k}}{g!(k)}$ is a normalisation constant [19, 28]. We here assume that *V*, $\lambda$ and $g(\cdot )$ are such that $z(\varphi (x))<\infty$ for all $x\in \Lambda _L$. This is in particular the case for any *V* and $\lambda$, when $g(\cdot )$ satisfies $g(k)\ge ck$ for some constant $c>0$ [28].

On restricting the model to a fixed number of particles *N*, the invariant measure $\pi$ (which is called the canonical measure) can be obtained by a conditioning of (57). Note that (57) has the structure of a product measure. Also if $g(k)=k$ then one recovers the case of non-interacting particles and the local marginals of (57) are Poisson distributions.

To make the comparison between reversible and irreversible models described in Sect. 2.1, we require an irreversible model whose invariant measure is (57). Again using that $\sum _\eta \pi (\eta ) \mathcal {L}f(\eta )=0$ for all *f*, we take $f=\eta (x)$ to be the number of particles on site *x*, from which we see that the irreversible rates (55) are also consistent with the invariant measure (57) if we take

58 $$\begin{aligned} \sum _{y:y\sim x} (k_{x,y}-k_{y,x})=0~\text { for all }x, \end{aligned}$$

where the notation $y\sim x$ indicates that sites *x* and *y* are nearest neighbours. (If we imagine a system with just one particle, this constraint states that the rate of hopping onto site *x* is balanced by the rate of hopping away from that site. For the ZRP, this same balance condition ensures that the invariant measure (57) is still valid even for many interacting particles).

Finally then, the conditions on the perturbations $k_{x,y}$ required for a meaningful comparison between reversible and irreversible models can be summarised as:

59 $$\begin{aligned} \sum _{y:y\sim x} k_{x,y}=0,~\text { }~k_{x,y}=-k_{y,x},~\text { and }~|k_{x,y}|< \mathrm e^{-(V(x)+V(y))/2}. \end{aligned}$$

The rates $k_{x,y}$ can be interpreted as elements of a matrix, which coincides (up to the factor 1 / 2) with the vorticity matrix $\Gamma$ introduced in [10].

In terms of the splitting (7) the symmetric part of the dynamics is given by $c_s(x \rightarrow y) = \mathrm{e}^{\frac{1}{2}[V(x)-V(y)]}$ and the anti-symmetric part by $c_a(x \rightarrow y) = k_{x,y} \mathrm{e}^{V(x)}$, such that the symmetric part (corresponding to $\mathcal L_S$) is independent of $k_{x,y}$.

#### Hydrodynamic Limit

The hydrodynamic limit of the ZRP is defined as follows. For a ZRP on a lattice $\Lambda _L$ with $L^d$ sites, one takes $N=\lfloor \rho _0 L^d\rfloor$ particles, where $\rho _0$ is the average density. The lattice $\Lambda _L$ is mapped into the domain $[0,1]^d$ by identifying each site $x\in \Lambda _L$ with a position $\tilde{x} \in \Lambda$ with $\Lambda = [0,1]^d$. Hence the site *x* with integer co-ordinates $(i,j,\dots )$ has a position $\tilde{x}=(i/L,j/L,\dots )$. Roughly speaking, the density $\rho _t(\tilde{x})$ in the MFT is equal to the typical number of particles on site *x*, and the normalisation of the density is $\int _\Lambda \rho _t(\tilde{x}) \;\! d\tilde{x}=\rho _0$. The hydrodynamic limit corresponds to a sequence of models in which $L\rightarrow \infty$ at fixed $\rho _0$, so $N\rightarrow \infty$.

The hydrodynamic limit corresponds to observing a system on increasingly large length and time scales. Note that since the number of sites in $\Lambda _L$ is diverging (proportional to $L^d$) in the hydrodynamic limit, the diffusion constant for a single particle (in $\Lambda$) vanishes as $L^{-2}$. For this reason, when the lattice $\Lambda _L$ is mapped into the fixed domain $\Lambda$, it is also convenient to scale the hop rates for all particles, by taking $c(x\rightarrow y)\rightarrow L^2 c(x\rightarrow y)$. This ensures that the diffusive behaviour characteristic of the hydrodynamic limit is observed, and the hydrodynamic limit is consistent with MFT.

To fix the hop rates between sites in the ZRP, one fixes a smooth potential function $\tilde{V} :\Lambda \rightarrow \mathbb {R}$ on the hydrodynamic scale, and one considers a sequence of ZRPs of increasing sizes *L* with potential functions $V(x)=\tilde{V}(\tilde{x})$, where $\tilde{x}$ is the image in $\Lambda$ of the discrete site $x\in \Lambda _L$. Similarly one fixes a vector field $\tilde{k} :\Lambda \rightarrow \mathbb {R}^d$ and takes $k_{x,y}=\tilde{k}(\tilde{x})\cdot (\tilde{y} - \tilde{x})$ where the dot indicates a scalar product in $\mathbb {R}^d$.

The relation between the ZRP and the MFT is discussed in e.g. [7, 22] and in the review paper [9]. In particular, for both reversible and irreversible ZRPs one arrives at the situation described in Sect. 2.2.5. The hydrodynamic limit (52) depends on the drift function $E:\Lambda \rightarrow \mathbb {R}^d$ which is given by $E(\tilde{x})=-\nabla \tilde{V}(\tilde{x})+\tilde{k}(\tilde{x})$.

The MFT description of the ZRP also depends on a function $\phi$ which can be obtained as the solution of

60 $$\begin{aligned} \rho = \sum _{k=1}^\infty \frac{k\;\!\phi (\rho )^{k}}{z(\phi (\rho )) g!(k)} . \end{aligned}$$

We identify the right hand side of this equation as the mean local density associated with the measure (57), at fugacity $\varphi =\phi (\rho )$.

The quasipotential $\mathcal V$ for the ZRP is given by [9],

61 $$\begin{aligned} \mathcal V(\rho ) = \int _\Lambda \biggl [\rho (x) \log \biggl (\frac{\phi (\rho (x))}{\phi (\bar{\rho }(x))}\biggr ) - \log \biggl (\frac{z(\phi (\rho (x)))}{z(\phi (\bar{\rho }(x)))}\biggr )\biggr ] dx. \end{aligned}$$

### Simulation Results

We present numerical results for one-dimensional and two-dimensional systems, showing how breaking detailed balance [that is, taking $k_{x,y}\ne 0$ in (55)] accelerates convergence to equilibrium. The simulations are performed using the Gillespie algorithm [21]. The results illustrate several aspects of the theoretical analysis in Sect. 2. First, the results of that section do not rely on how detailed balance is broken: we show that there are several possible choices and discuss their consequences. Second, our numerical results show in what contexts we expect to see significant acceleration of the dynamics on breaking detailed balance, and in what contexts we expect the acceleration to be mild.

In all cases, we show results that are scaled to be consistent with the hydrodynamic limit. That is, we map the lattice $\Lambda _L$ into $[0,1]^d$ and we rescale the microscopic hop rates by a factor of $L^2$ so as to recover diffusive behaviour in the hydrodynamic limit.

In practical situations where the rate of convergence to equilibrium is important, a common situation is that the potential function *V* is not convex, but includes several (or many) minima, separated by high barriers. From a physical perspective, the temperature of our systems is a parameter that has been absorbed into the function *V*. In general, high barriers are linked with long (Arrhenius) time scales that are proportional to $\mathrm{e}^{\Delta V}$. In order to understand whether breaking detailed balance can accelerate convergence in such non-convex problems, we consider cases where the function *V* has two minima, with longest time scale in the system corresponding to motion between these minima.

#### Characterisation of Convergence

We perform numerical simulations starting from a fixed (deterministic) initial condition $\eta _0$. To analyse convergence to equilibrium, we perform numerical simulations of the ZRP, and we track the time-dependence of several different quantities. For any configuration $\eta$, the mean potential energy is

62 $$\begin{aligned} \langle \eta , V\rangle = \sum _{x\in \Lambda _L} \eta (x) V(x). \end{aligned}$$

We generate several trajectories (sample paths) $\eta _t$ of the ZRP and we estimate the mean potential energy

63 $$\begin{aligned} {\hat{V}}(t)=\mathbb {E}_{\mu _0}(\langle \eta _t, V\rangle ) \end{aligned}$$

by taking the mean value of $\langle \eta _t, V\rangle$ over these trajectories. For systems of non-interacting particles (where $\phi (\rho )=\rho$), we also estimate the macroscopic relative entropy as

64 $$\begin{aligned} D(t) = \sum _{x\in \Lambda _L} \mathbb {E}_{\mu _0}(\eta _t(x)) \log \biggl (\frac{\mathbb {E}_{\mu _0}(\eta _t(x))}{\mathbb {E}_\pi (\eta (x))}\biggr ), \end{aligned}$$

which can be seen as an approximation to the quasipotential, which is for an independent random walk given by

$$\begin{aligned} \mathcal V(\rho _t) = \int _\Lambda \biggl [\rho _t(x) \log \Bigl (\frac{\rho _t(x)}{\bar{\rho }(x)}\Bigr ) + \rho _t(x) - \bar{\rho }(x) \biggr ]\;\!dx =\int _\Lambda \rho _t(x) \log \Bigl (\frac{\rho _t(x)}{\bar{\rho }(x)}\Bigr ) \;\!dx, \end{aligned}$$

where we used the fact that $z(\varphi ) = \mathrm e^{-\varphi }$ in (61) and the last identity follows from the fact that the density is conserved: $\int _\Lambda \rho _t(x) dx = \int _\Lambda \bar{\rho }(x) dx$.

For numerical purposes, we estimate $\mathbb {E}_{\mu _0}(\eta _t(x))$ as the average occupancy of site *x* over the sample paths that we generate, and we calculate $\mathbb {E}_\pi (\eta (x))$ by direct construction of the invariant measure (whenever possible). Finally, we estimate the Gibbs entropy

65 $$\begin{aligned} S(t) = -\sum _x \mathbb {E}_{\mu _0}(\eta _t(x)) \log \mathbb {E}_{\mu _0}(\eta _t(x)), \end{aligned}$$

which is large if particles are delocalised throughout the system, and small if they are concentrated on a small number of sites. Again, we estimate $\mathbb {E}_{\mu _0}(\eta _t(x))$ as the average occupancy of site *x* over the sample paths that we generate, which provides an estimator of *S*.

These three quantities ${\hat{V}},D,S$ all converge as a function of time to stationary values, providing differing information as to the rates of convergence. Note that for non-interacting particles, $\overline{\rho }(x)=\mathbb {E}_\pi (\eta (x))=\mathrm{e}^{-V(x)}/z$ for some constant *z*, so $D(t) = -S(t)+\hat{V}(t) + \log z$.

#### One-Dimensional Case: Results

We consider periodic boundaries for a model on a one-dimensional strip, this is equivalent to motion on the perimeter of a circle (flat torus in one dimension). In this case condition (59) requires $k_{x,x+1} =k_{x-1,x}$, so we set $k_{x,x+1}=c$ with some constant *c* that is independent of *x*. The choice $c>0$ corresponds to a fixed force $c\;\!\mathrm e^{V}$ that is forcing the particles to travel around the circle. For a hydrodynamic limit consistent with macroscopic fluctuation theory, we require *c* to vary with the system size *L* as $c=E/L$ with *E* a fixed constant [9].

We note in passing that the use of periodic boundaries is essential for breaking balance in these closed systems: on a finite strip with reflecting boundary conditions, (59) has no solutions except $k_{x,y}=0$ so there is no way to break detailed balance.

Thus, returning to the case with the periodic boundaries, the generator is

66 $$\begin{aligned} \mathcal L f(\eta ) = \sum _{x=0}^{L-1} \Bigl [\bigl (f(\eta ^{x,x+1})-f(\eta )\bigr )\;\!L^2g(\eta (x))\bigl ( \mathrm e^{(V(x)-V(x+1))/2} + {(E/L)} \mathrm e^{V(x)}\bigr )\\ +\bigl (f(\eta ^{x,x-1})-f(\eta )\bigr )\;\!L^2g(\eta (x))\bigl ( \mathrm e^{(V(x)-V(x-1))/2} - {(E/L)} \mathrm e^{V(x)}\bigr )\Bigr ], \end{aligned}$$

where the addition is periodically extended on $\Lambda _L=\{0,\dots ,L-1\}$, i.e., $(L-1)+1=0$ and $0-1=L-1$. We take $g(k)=k$ so that the particles do not interact. The potential is

67 $$\begin{aligned} V(x)=A \sin (4\pi x/L)- B \cos (2\pi x/L) \end{aligned}$$

with $A=3/2$ and $B=3/4$ so that the global minimum of the potential is at ${\hat{x}} \approx 0.888$ with $V \approx -2.052$. The height of the barrier is approx 2.609. The initial condition has all particles on a single site, $x_0=L/4$, in the vicinity of the secondary minimum. The stationary state has $\overline{\rho }(x)=\mathbb {E}_\pi (\eta (x)) \propto \mathrm{e}^{-V(x)}$ with a proportionality constant determined by the total density (which in this case is $z\approx 2\;\!377)$. The parameter *E* in Eq. (66) is set to $E=36$. For the lattice size $L=300$, the maximal value allowed for *E* to ensure that $c_s+c_a\ge 0$ is slightly above 38.4. In principle one can choose larger values for *E* by increasing the lattice size *L*.

**Fig. 1:**
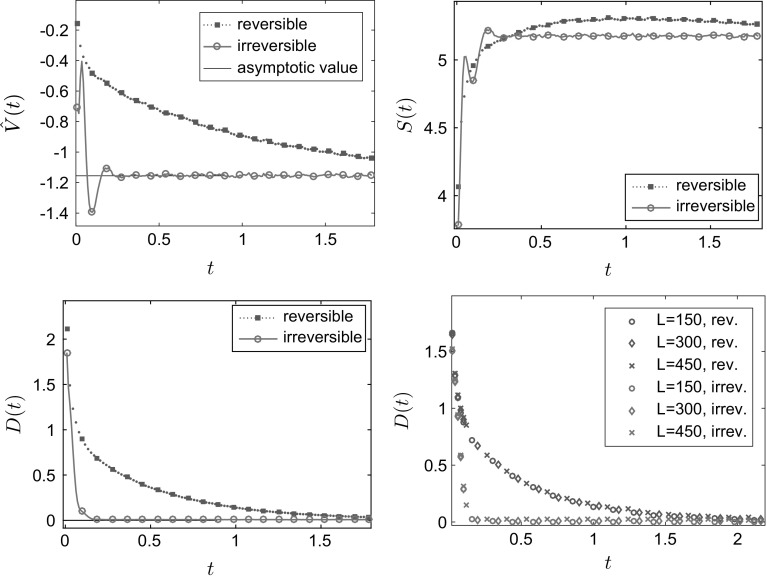
One-dimensional simulation for independent particles on a *circle* with $L=300$ sites, comparing reversible and irreversible drift-diffusion processes as described in the main text with the potential (67). *Top row and bottom left* plot of the test observables average energy $\hat{V}$, Gibbs entropy *S* and relative entropy *D* for $E=36$. *Bottom right* plot of the relative entropy *D* for different system sizes $L=150,300,450$, all for $E=18$. As predicted by the hydrodynamic equation, varying the system size at fixed *E* and rescaling time by a factor of $L^2$ leads to limiting behaviour independent of *L*. All results were obtained by averaging over 20, 000 individual particle trajectories

**Fig. 2:**
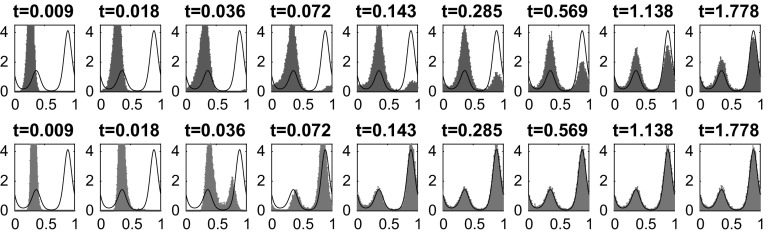
One-dimensional simulation for independent random walk on a *circle* with the potential (67). Configuration at different times for the reversible (*top row*) and the irreversible (*bottom row*) process with drift ‘to the right’ and steady state (*in black*). *x*-axis: position. *y*-axis: averaged number of particles. In the irreversible case, $E=36$

The results in Fig. 1 are for a domain of size $L=300$; we also compared this to simulations for $L=150$, $L=300$ and $L=450$ for the value $E=18$ (to ensure positiveness of the transition rates for $L=150$). We found the results to be qualitatively very similar, see the bottom right panel in Fig. 1. Figure 1 shows the convergence to equilibrium of the mean potential energy and the entropy. One sees that convergence of both the energy and the entropy is significantly faster when detailed balance is broken. To illustrate the mechanism for this effect, Fig. 2 shows how the mean density $\mathbb {E}_{\mu _0}(\eta _t(x))$ varies with time. In the irreversible case, the non-gradient part of the drift force *E* acts to the right and is equal to $c\;\!\mathrm{e}^V$, so it is large near the maxima of the potential. This prevents the system from becoming localised in the secondary (local) minimum and aids convergence to the steady state. By contrast, in the reversible system, the particles need to *diffuse* over the maxima of the potential, which is a slower process. This difference explains the much faster convergence to the steady state observed in Fig. 1. The overshoot of the entropy for the reversible case in Fig. 1 occurs because the state where the particles are distributed evenly between the two minima has a higher entropy *S* than the steady state (where they are localised primarily in the global minimum). The state where the particles are distributed evenly between the minima is an example of a situation where the gradient of the free energy is small (within the relevant metric), so that steepest descent of the free energy leads to slow changes in the density.

Note also that (64) implies that $D(t)\rightarrow 0$ at long times, as the system converges to its steady state. However, in Fig. 1 one sees that our estimate of *D*(*t*) converges instead to a small positive constant. This offset arises because our estimator of *D*(*t*) is biased: it is based on *m* independent numerical simulations (each with *N* particles) and the expectation value of our estimator converges to *D*(*t*) only as $m\rightarrow \infty$. Specifically, we estimate $\mathbb E_{\mu _0}(\eta _t(x))$ as $\vartheta _t(x) = m^{-1}\sum _{k=1}^m\eta ^k_t(x)$ where $\eta ^k_t(x)$ is the number of particles on site *x* at time *t* in the *k*-th simulation. Inserting this estimate into the (nonlinear) expression (64), it is easily shown that the resulting estimator of *D*(*t*) has in general a finite bias. However, as $m\rightarrow \infty$, $\vartheta$ obeys a law of large numbers and converges almost surely to $\mathbb E_{\mu _0}(\eta _t(x))$—hence our estimator converges to *D*(*t*) as $m\rightarrow \infty$.

#### One-Dimensional Case: Discussion

This one-dimensional model is useful for illustrative purposes and establishes the general principles derived in Sect. 2. However, the restriction to one dimension means that detailed balance can only be broken by applying a driving force $c\,\mathrm{e}^{V}$ (otherwise the invariant measure would be changed). If barriers are large, one sees that the driving force near the top of the barrier must be very large indeed: it is hard to see how this can be realised in practical applications. Physically, the idea is to drive a constant current around the periodic system, and this requires the drift velocities (and hence forces) to be largest at the top of any barriers, where the density is least. In this sense, it is perhaps not surprising that by applying large forces to quickly drive particles over all barriers in the system, one can significantly speed up mixing of the particles between the two minima of the potential.

For these reasons, we turn to a two-dimensional system, where there are many more ways of breaking detailed balance while preserving the same invariant measure.

#### Two Dimensional Case: Model and Results

In two dimensions, there is considerably more freedom in the choice of the rates $k_{x,y}$. If one again assumes periodic boundaries, it is always possible to have all non-gradient forces acting in a single direction: for example $k_{x,x+e_1}=c$ where $e_1$ is a lattice vector, as in the previous one-dimensional example. However, this requires driving forces that depend exponentially on the value of the potential, as in one dimension. We therefore pursue a different strategy.

**Fig. 3:**
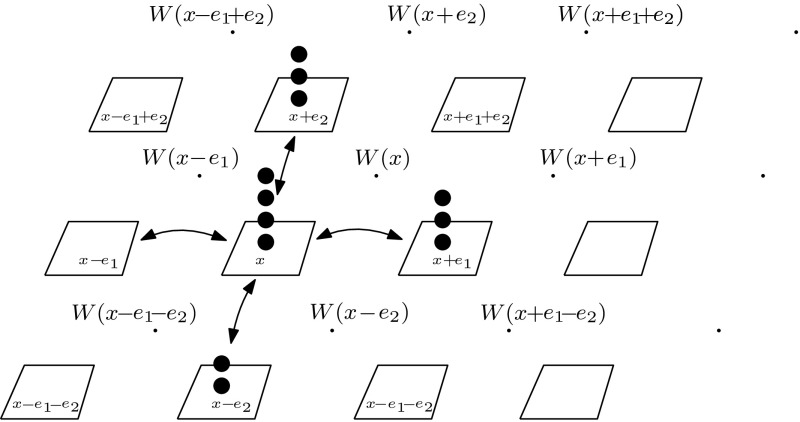
The function *W* defined on the plaquettes; $e_1$ and $e_2$ are the Euclidean basis vectors

Denoting the Euclidean basis for $\Lambda _L$ with $e_1,e_2$, Eq. (59) implies that both $k_{x,x\pm e_j}= -k_{x\pm e_j,x}$ and $k_{x,x+e_1}+k_{x,x-e_2}+k_{x,x-e_1}+k_{x,x+e_2} =0$ have to be satisfied. One way to choose appropriate $k_{x,y}$ is to consider the plaquettes of the square lattice as in Fig. 3 and to define a *vorticity* *W* at the centre of each plaquette. The value of *W* on the plaquette centred at $x+\frac{1}{2}(e_1+e_2)$ is *W*(*x*). One then can choose the rates $k_{x,y}$ as the following differences:

68 $$\begin{aligned} k_{x,x+e_1}&= W(x\!-\!e_2)-W(x) \nonumber \\ k_{x,x-e_2}&= W(x\!-\!e_1\!-\!e_2)-W(x\!-\!e_2) \nonumber \\ k_{x,x-e_1}&= W(x\!-\!e_1)-W(x\!-\!e_1\!-\!e_2) \nonumber \\ k_{x,x+e_2}&= W(x)-W(x\!-\!e_1) \end{aligned}$$

This choice satisfies both conditions $k_{x,y}=-k_{y,x}$ and $\sum _y k_{x,y}=0$. The quantity *W* can be identified as a vorticity, in the sense that taking $W(x)=W_0\delta _{x_0,x}$ with $W_0>0$ causes particles to circulate clockwise around plaquette $x_0$.

Any choice of the function *W* is possible, and should lead to acceleration of the dynamics, following the theoretical analysis of Sect. 2. Here we concentrate on a case where *W* is related to the potential *V*, so that the rates $c(x\rightarrow y)$ depend only on the gradients of the potential in the vicinity of site *x*. (The physical idea is that particle motion is naturally sensitive to local potential gradients since these correspond to forces acting on the particles. On the other hand, the motion of a particular particle should not be sensitive to the total energy *V*, since this depends on the state of the system far away from that particle). To arrive at forces that depend only on potential gradients, we take $W(x)=a\cdot \exp (\frac{1}{4}[V(x)+V(x+e_1)+V(x+e_2)+V(x+e_1+e_2)])$, where *a* is a parameter that sets the scale of the vorticity.

On taking the hydrodynamic limit, this gives rise to the driving force

69 $$\begin{aligned} E({\tilde{x}}) = -\nabla {\tilde{V}}({\tilde{x}}) + a[ e_{1} \nabla _2 {\tilde{V}}({\tilde{x}}) - e_{2} \nabla _1 {\tilde{V}}({\tilde{x}})] , \end{aligned}$$

where $a>0$ (recall from Sect. 3.1.3 that $\tilde{x}$ is the image in $\Lambda$ of the discrete site $x\in \Lambda _{L}$). We recognise the second term on the right hand side as a force that is obtained by rotating $\nabla V$ clockwise by $\pi /2$ radians, so that it acts to drive the system around the level sets of *V*.

**Fig. 4:**
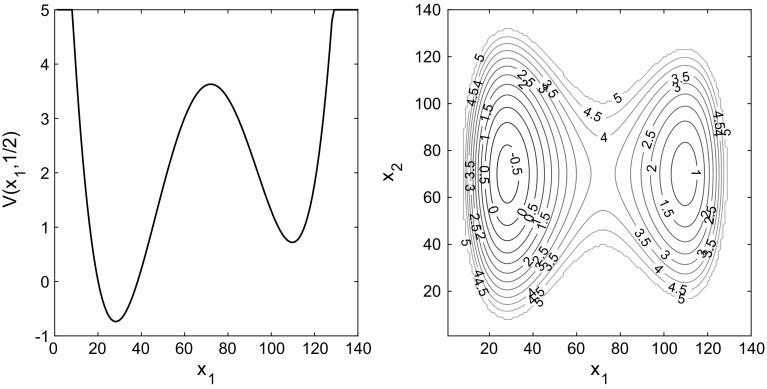
*Left* $x_1$-cross-section of *V*(*x*) as given in (70) for $x_2=1/2$. *Right* level sets of *V*(*x*)

**Fig. 5:**
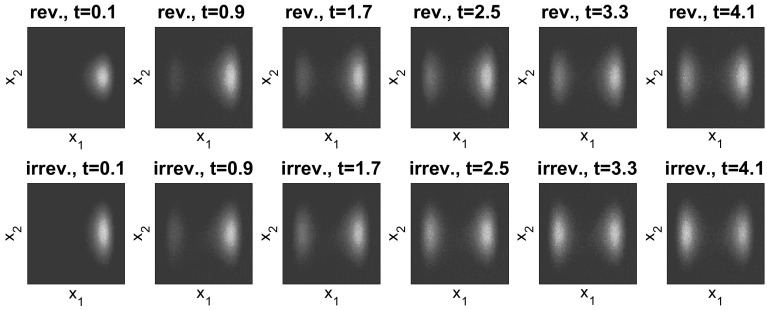
Configuration for $g(k)=k$ with the potential (70) at different times. (*Dark*) *blue* means low number of particles, *yellow* means many particles. *Top row* reversible process. *Bottom row* irreversible process (Color figure online)

**Fig. 6:**
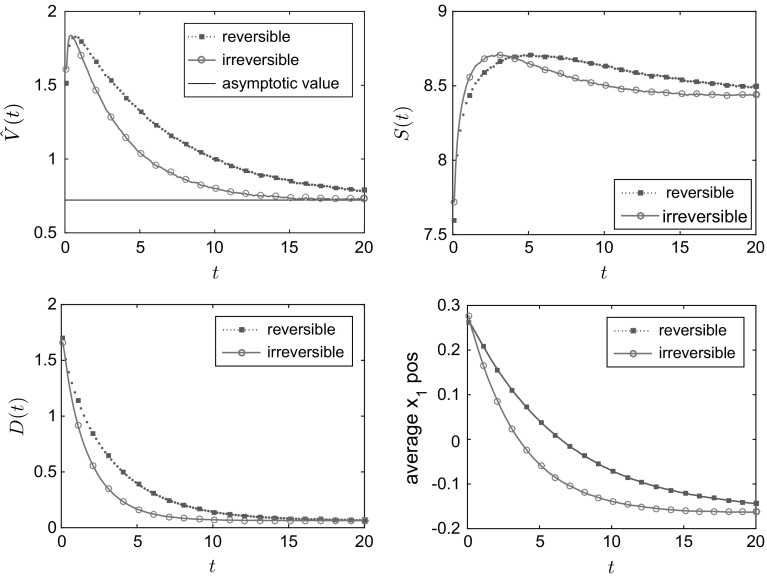
ZRP with $g(k)=k$ (independent random walk) with initial position of all particles in the local minimum. Average energy ${\hat{V}}$, the Gibbs entropy *S*, the relative entropy *D* and the average $x_1$-position of particles. The initial position of the particle is at a fixed position in the local (but not global) minimum of the potential (70). The domain size is $L^2=140^2$ and we averaged over 16 simulations consisting of 9800 particles each

The following simulations are on a two dimensional closed domain with $L=140$ and zero flux at the boundary, i.e., the domain has $140\times 140=19\;\!600$ sites and the particles cannot leave the domain.

**Fig. 7:**
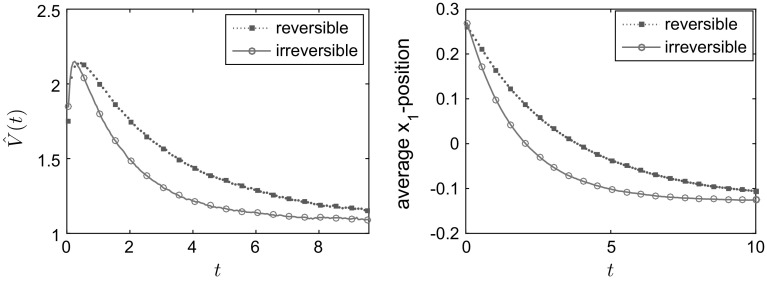
ZRP with $g(k)=k^{3/2}$ and the particles are started in the local minimum. *Left* average energy ${\hat{V}}$. *Right* average $x_1$-position

**Fig. 8:**
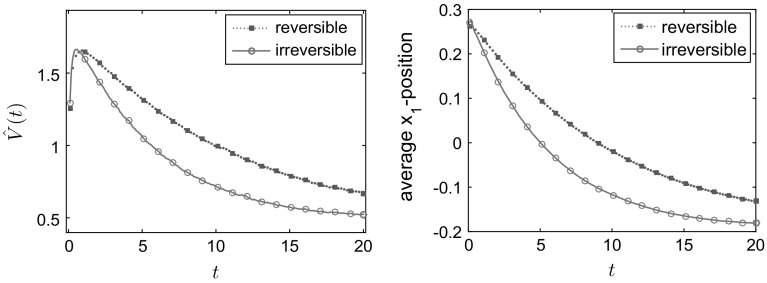
ZRP with $g(k)=k^{5/6}$ and the particles are started in the local minimum. We again plot the average energy ${\hat{V}}$ and the average $x_1$-position

We consider three different ZRPs, corresponding to different choices for *g*(*k*). Firstly, we consider the linear case (independent particles), where $g(k)=k$. We further consider the superlinear case with $g(k)=k^{3/2}$, such that the particles repel each other (the hop rates away from site *x* is increased when that site contains more particles). Finally we investigate the sublinear case with $g(k) = k^{5/6}$ in which the particles prefer to cluster together. For each setting, we simulated the process with both reversible and irreversible dynamics, with $L^2/2=9\;\!800$ particles averaged over 16 simulations. The potential, which is also depicted in Fig. 4, is for shifted coordinates $x=(x_1,x_2)\in [-1/2,1/2]^2$ given by

70 $$\begin{aligned} V(x_1,x_2) = A(x_1^2 - B)^2 + C x_2^2 + D x_1 \end{aligned}$$

with a cut-off at a given height $V^*$. For the simulations we chose the parameters $A=500$, $B=0.085$, $C=30$, $D=2.5$ and $V^*=5$ (that is, the potential used is $\mathrm{max}(V(x_1,x_2),V^*)$). The parameter in (69), which sets the strength of the non-gradient term of the driving force, was set to $a=0.4$. This value is again close to the maximal allowed value (which is slightly above 0.405).

For all simulations, the particles start at position $(0.5,0.75)\in [0,1]^2$ close to the local minimum of the double well potential. The particles then try to leave this well and move to the global minimum (on the left) as can be seen in the plots in Fig. 5 for the linear case. The test observables for the linear/superlinear/sublinear case can be found in Figs. 6, 7 and 8, respectively. Depending on the chosen configuration, the simulation time on a HPC node with 16 cores using Matlab took between 10 and 13.5 hours.

As in the one-dimensional case, the particles are under the irreversible dynamics able to leave the minimum faster than it is the case for reversible dynamics (compare the bottom row with the top row in Fig. 5).

**Table 1:** Table of the absolute times $t_s$ for the reversible process (left) and ratios between times of the reversible and irreversible process $t_s/t_a$ (right) to reach the distances $\Delta {\hat{V}}=0.3$ and $\Delta x_1=0.2$, respectively

$t_s$	$\Delta \hat{V}$	$\Delta x_1$	$t_s/t_a$	$\Delta \hat{V}$	$\Delta x_1$
$g(k) = k$	2.38	1.30	$g(k) = k$	1.83	1.79
$g(k) = k^{3/2}$	1.11	0.58	$g(k) = k^{3/2}$	1.78	1.86
$g(k) = k^{5/6}$	3.55	2.03	$g(k) = k^{5/6}$	1.77	1.80

#### Two Dimensional Case: Discussion

We close this section with Table 1, which quantifies the acceleration in the models where particles attract, repel, or have no interactions. For this, we consider the average energy ${\hat{V}}$ and the average $x_1$ position of the particles. Assuming that the final values of these observables in the irreversible simulations are close to their steady-state values, we consider the distance $\Delta {\hat{V}}$ (resp. $\Delta x_1$) of both the reversible and irreversible process and keep track of the first time where the distance is below a given threshold. Denoting this time for the reversible process with $t_s$ and for the irreversible process with $t_a$, we can use the ratio $t_s/t_a$ as an estimator for the acceleration.

From the data in the table, on sees that the processes are typically accelerated by factors about 1.75 independent of the choice of *g*(*k*). We checked different thresholds (here we displayed $\Delta \hat{V}=0.3$ and $\Delta x_1=0.2$) which all lead to the same conclusions.

These are significant accelerations, although considerably less than the dramatic speedup of order 10 observed in one dimension. However, the physical mechanisms for the acceleration are different in the two cases. In one dimension, the drift forces which act to push particles up and over the barrier, so the forces are very large at the top of the barrier. In two dimensions, the effect is more subtle: returning to Fig. 4 and recalling that the drift force in (69) is obtained by a rotation of the potential gradient, one sees that in the vicinity of the saddle point of the potential, there is a net drift to the left in the top part of Fig. 4b, and a drift to the right in the bottom part. A natural analogy is a gentle stirring motion that happens in the vicinity of the saddle point, and tends to accelerate mixing. This seems a much more plausible mechanism for accelerating convergence to equilibrium in practical situations, compared with the large forces required in one dimension.

Finally, we note that transport between the minima of a non-convex potential energy always involves a slow time scale proportional to $\mathrm{e}^{\Delta V}$, since a particle must still reach the barrier in order to cross it, and the probability that a particle visits the barrier is proportional to $\mathrm{e}^{-\Delta V}$. However, the results here show that mixing of particles between energy minima can be accelerated by enhancing the probability that if a particle reaches a region with high *V*, it takes advantage of this excursion in order to cross the barrier. The mechanisms for this enhanced probability differ between the models considered here—it would be interesting to investigate this effect further, so as to understand how general these mechanisms are and how they can be exploited in practical applications.

## Conclusion and Outlook

We have considered interacting particle systems described by Markov chains, and their hydrodynamic limits, as described by macroscopic fluctuation theory. We compare reversible and irreversible processes: for an irreversible system with generator $\mathcal {L}$, the corresponding reversible process is the one identified in (7), whose generator is $\mathcal {L}_S$. At the microscopic level, it is known that the irreversible process then converges to its steady state at least as fast as the reversible one—this can be demonstrated by considering either the spectral gap or the (level-2) large deviations of the empirical measure. In the hydrodynamic limit, Eq. (38) shows that this property is preserved, by considering the large deviations of the empirical density. Moreover, Eq. (51) gives a quantitative expression for the acceleration of convergence, which may be seen as a generalisation of previous results for single-particle diffusions [35].

Our numerical results for the ZRP reinforce the observation that for a given reversible system, there is a large family of irreversible systems for which convergence to equilibrium is faster (or, at least, equally fast). We considered two cases: either a drift force in a single direction, which acts to drive a system around a circle (Sect. 3.2.2) or the introduction of a force that drives the system around the level sets of the potential (Sect. 3.2.4). In both cases, we observe acceleration of convergence, as expected.

The results within MFT provide a geometrical interpretation of the acceleration, in terms of forces that act in directions perpendicular to the free energy gradient, as shown by orthogonality relations for currents such as Eq. (46). We have argued that such forces can act to accelerate convergence by driving the system away from regions where the free energy gradient is shallow, in which cases reversible processes exhibit slow convergence.

We offer two perspectives on future application of these ideas. First, we have shown that breaking detailed balance generically accelerates convergence, but of course there are very many ways to write down irreversible models, and it is not clear what choices are most practical in applications, nor which ones lead to the fastest convergence. In particular, the choice considered for ZRP examples shown here are rather specific to systems in one or two dimensions. (We emphasise however that the configuration spaces of the ZRP are very high-dimensional since we consider *N* interacting particles, so the methods are not restricted to systems with low-dimensional configuration spaces.) Second, we gave a geometrical interpretation in which the symmetric dynamics correspond to the gradient flow (steepest descent) of the free energy and the antisymmetric dynamics are in some sense orthogonal to this gradient flow. This offers a potentially new perspective on hydrodynamic limits in irreversible systems, which it would be interesting to investigate further, for example with a view towards obtaining analytic estimates for the rate of convergence.

Supporting data for this manuscript and the code used for the simulations will be made available short after publication on the University of Bath data archive (DOI:10.15125/BATH-00365).

## References

[1] Adams, S., Dirr, N., Peletier, M., Zimmer, J.: Large deviations and gradient flows. Philos. Trans. R. Soc. Lond. Ser. A Math. Phys. Eng. Sci. **371**(2005), 20120341, 17 (2013) 10.1098/rsta.2012.0341 24249769

[2] Asmussen S, Glynn PW (2007). Stochastic Simulation: Algorithms and Analysis, Stochastic Modelling and Applied Probability.

[3] Baiesi, M., Maes, C., Wynants, B.: Fluctuations and response of nonequilibrium states. Phys. Rev. Lett. **103**, 010602 (2009) 10.1103/PhysRevLett.103.010602 19659132

[4] Barato AC, Chetrite R (2015). A formal view on level 2.5 large deviations and fluctuation relations. J. Stat. Phys..

[5] Bernard EP, Krauth W, Wilson DB (2009). Event-chain Monte Carlo algorithms for hard-sphere systems. Phys. Rev. E.

[6] Bertini, L., De Sole, A., Gabrielli, D., Jona-Lasinio, G., Landim, C.: Fluctuations in stationary nonequilibrium states of irreversible processes. Phys. Rev. Lett. **87**(4), 040601 (2001) 10.1103/PhysRevLett.87.040601 11461605

[7] Bertini L, De Sole A, Gabrielli D, Jona-Lasinio G, Landim C (2002). Macroscopic fluctuation theory for stationary non-equilibrium states. J. Stat. Phys..

[8] Bertini L, Faggionato A, Gabrielli D (2015). Large deviations of the empirical flow for continuous time Markov chains. Ann. Inst. Henri Poincaré Probab. Stat..

[9] Bertini L, De Sole A, Gabrielli D, Jona-Lasinio G, Landim C (2015). Macroscopic fluctuation theory. Rev. Mod. Phys..

[10] Bierkens J (2015). Non-reversible Metropolis-Hastings. Stat. Comput..

[11] Bodineau T, Derrida B (2004). Current fluctuations in nonequilibrium diffusive systems: an additivity principle. Phys. Rev. Lett..

[12] Chen, F., Lovász, L., Pak, I.: Lifting Markov chains to speed up mixing. In: Annual ACM Symposium on Theory of Computing (Atlanta, GA, 1999), pp. 275–281 (electronic). ACM, New York (1999)

[13] Derrida, B.: Non-equilibrium steady states: fluctuations and large deviations of the density and of the current. J. Stat. Mech. Theory Exp. **2007**(7), P07023 (electronic) (2007)

[14] Diaconis P (2009). The Markov chain Monte Carlo revolution. Bull. Am. Math. Soc. (N.S.).

[15] Diaconis P, Holmes S, Neal RM (2000). Analysis of a nonreversible Markov chain sampler. Ann. Appl. Probab..

[16] Diaconis P, Saloff-Coste L (1996). Logarithmic Sobolev inequalities for finite Markov chains. Ann. Appl. Probab..

[17] Donsker MD, Varadhan SRS (1983). Asymptotic evaluation of certain Markov process expectations for large time IV. Commun. Pure Appl. Math..

[18] Duncan AB, Lelièvre T, Pavliotis GA (2016). Variance reduction using nonreversible Langevin samplers. J. Stat. Phys..

[19] Evans MR, Hanney T (2005). Nonequilibrium statistical mechanics of the zero-range process and related models. J. Phys. A.

[20] Gilbarg, D., Trudinger, N.S.: Elliptic partial differential equations of second order. Class. Math. Springer, Berlin (2001). Reprint of the 1998 edition

[21] Gillespie DT (1977). Exact stochastic simulation of coupled chemical reactions. J. Phys. Chem..

[22] Hirschberg, O., Mukamel, D., Schütz, G.M.: Density profiles, dynamics, and condensation in the ZRP conditioned on an atypical current. J. Stat. Mech. Theory Exp. **2015**(11), P11023 (2015)

[23] den Hollander F (2000). Large Deviations, Fields Institute Monographs.

[24] Hwang CR, Hwang-Ma SY, Sheu SJ (2005). Accelerating diffusions. Ann. Appl. Probab..

[25] Hwang CR, Normand R, Wu SJ (2015). Variance reduction for diffusions. Stoch. Process. Appl..

[26] Ichiki A, Ohzeki M (2013). Violation of detailed balance accelerates relaxation. Phys. Rev. E.

[27] Jack R, Zimmer J (2014). Geometrical interpretation of fluctuating hydrodynamics in diffusive systems. J. Phys. A: Math. Theor..

[28] Kipnis, C., Landim, C.: Scaling limits of interacting particle systems. Grundlehren der Mathematischen Wissenschaften [Fundamental Principles of Mathematical Sciences], vol. 320. Springer, Berlin (1999)

[29] Labbé, C., Lacoin, H.: Cutoff phenomenon for the asymmetric simple exclusion process and the biased card shuffling. arXiv preprint arXiv:1610.07383 (2016)

[30] Lelièvre T, Nier F, Pavliotis GA (2013). Optimal non-reversible linear drift for the convergence to equilibrium of a diffusion. J. Stat. Phys..

[31] Levin, D.A., Peres, Y., Wilmer, E.L.: Markov chains and mixing times. American Mathematical Society, Providence, RI (2009). With a chapter by James G. Propp and David B. Wilson

[32] Liggett, T.M.: Interacting particle systems. Classics in Mathematics. Springer, Berlin (2005). Reprint of the 1985 original

[33] Maas J (2011). Gradient flows of the entropy for finite Markov chains. J. Funct. Anal..

[34] Newman MEJ, Barkema GT (1999). Monte Carlo methods in statistical physics.

[35] Rey-Bellet L, Spiliopoulos K (2015). Irreversible Langevin samplers and variance reduction: a large deviations approach. Nonlinearity.

[36] Rey-Bellet L, Spiliopoulos K (2016). Improving the convergence of reversible samplers. J. Stat. Phys..

[37] Sakai Y, Hukushima K (2016). Eigenvalue analysis of an irreversible random walk with skew detailed balance conditions. Phys. Rev. E.

[38] Spitzer F (1970). Interaction of Markov processes. Adv. Math..

[39] Sun, Y., Schmidhuber, J., Gomez, F.J.: Improving the asymptotic performance of Markov Chain Monte-Carlo by inserting vortices. In: Lafferty, J., Williams, C., Shawe-Taylor, J., Zemel, R.S., Culotta, A. (eds.) Advances in Neural Information Processing Systems 23, pp. 2235–2243. Neural Information Processing Systems Foundation (2010)

[40] Touchette H (2009). The large deviation approach to statistical mechanics. Phys. Rep..

